# PKM2, function and expression and regulation

**DOI:** 10.1186/s13578-019-0317-8

**Published:** 2019-06-26

**Authors:** Ze Zhang, Xinyue Deng, Yuanda Liu, Yahui Liu, Liankun Sun, Fangfang Chen

**Affiliations:** 1 Department of General Surgery, The First Hospital of Jilin University, Changchun, 130021 China; 2 Department of Pathophysiology, College of Basic Medical Sciences, Jilin University, Changchun, 130021 China; 3 Department of Gastrointestinal Surgery, The Second Hospital of Jilin University, Changchun, 130041 China; 4 Department of Gastrointestinal Colorectal and Anal Surgery, China-Japan Union Hospital of Jilin University, Changchun, 130021 China

**Keywords:** Cancer metabolism, Glycolysis, Pyruvate kinase, Warburg effect

## Abstract

Pyruvate kinase (PK), as one of the key enzymes for glycolysis, can encode four different subtypes from two groups of genes, although the M2 subtype PKM2 is expressed mainly during embryonic development in normal humans, and is closely related to tissue repair and regeneration, with the deepening of research, the role of PKM2 in tumor tissue has received increasing attention. PKM2 can be aggregated into tetrameric and dimeric forms, PKM2 in the dimer state can enter the nuclear to regulate gene expression, the transformation between them can play an important role in tumor cell energy supply, epithelial–mesenchymal transition (EMT), invasion and metastasis and cell proliferation. We will use the switching effect of PKM2 in glucose metabolism as the entry point to expand and enrich the Warburg effect. In addition, PKM2 can also regulate each other with various proteins by phosphorylation, acetylation and other modifications, mediate the different intracellular localization of PKM2 and then exert specific biological functions. In this paper, we will illustrate each of these points.

**Electronic supplementary material:**

The online version of this article (10.1186/s13578-019-0317-8) contains supplementary material, which is available to authorized users.

## Introduction

At the beginning of the twentieth century, German scientist Warburg discovered that in tumor tissues, even if oxygen is sufficient, malignant tumor cells still undergo active glucose glycolysis, the metabolic characteristic of this aerobic glycolysis is called Warburg effect [1]. And it is characterized by high glucose uptake rate, active glycolysis, and decrease of local microenvironment pH [2]. Pyruvate kinase (PK), as one of the key enzymes of glycolysis, acts on its substrate phosphoenolpyruvate (PEP) to form pyruvate [3], and pyruvate kinase (PK) has four different subtypes: L, R, M1, M2 [4]. PKL isoforms are mainly found in liver, kidney and red blood cells; while PKR is mainly expressed in red blood cells, biological activity is not clear; PKM1 is distributed in myocardium, skeletal muscle and brain tissue; PKM2 is distributed in tissues such as brain and liver [5]. Although the PKM2 tetramer and dimer are composed of the same monomer [6], the biological effects between the tetramer and the dimer are significantly different [7]. The tetramer mainly plays the role of pyruvate kinase and regulates the glycolysis and the dimer PKM2 in the context of glucose metabolism can be used as a switch for energy metabolism and material synthesis [8], routing glucose metabolism to pyruvate into the tricarboxylic acid cycle, converting to the pentose phosphate pathway, the uronic acid pathway, and the polyol pathway. In turn, a carbon source and a oxidation–reduction (REDOX) equivalent are provided for quinochrome ribose anabolism and non-essential amino acid anabolism [9, 10]. If in the context of non-glucose metabolism, after the tetramer is converted into a dimer, PKM2 can exist in a variety of different intracellular localizations, enter the nuclear to regulate gene expression, and attaches to the mitochondrial outer membrane to maintain mitochondrial function and localizes to the endoplasmic reticulum to inhibit endoplasmic reticulum stress [11]. Once again, PKM2 can also be modified with phosphorylation, acetylation and other proteins to regulate protein activity and intracellular localization. PKM2 can increase or even replace the original PK form regardless of the tissue-derived cells. Therefore, some researchers refer to PKM2 as tumor-specific PK [12].

## PKM2 dimer, tetramer and glucose metabolism

**Fig. 1:**
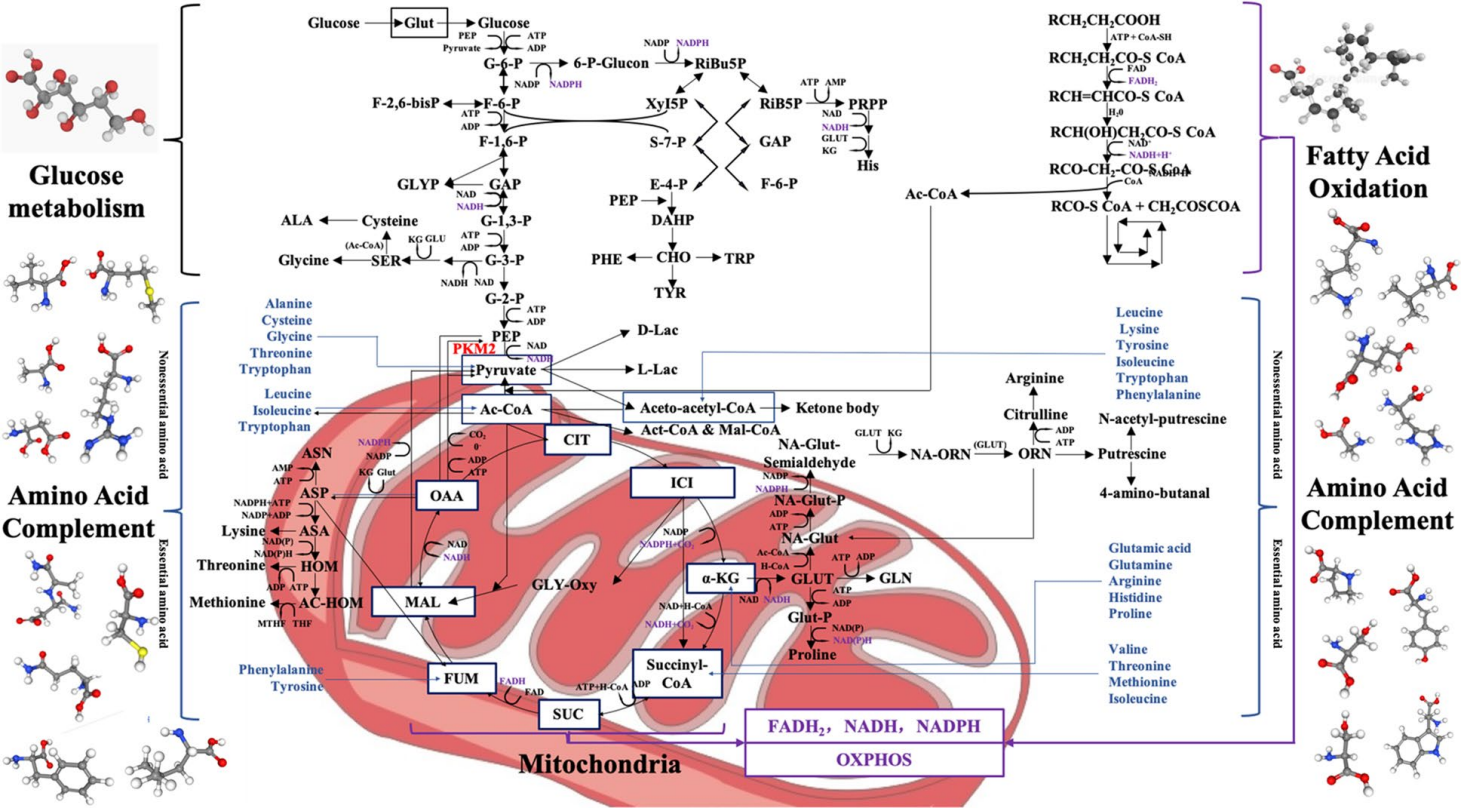
PKM2: Junction of Metabolic Networks and Signal Cascades. POST-Warburg effect, in glucose metabolism, tumor cells divide glucose metabolism into three separate parts of glycolysis, tricarboxylic acid cycle and oxidative phosphorylation (OXPHOS). The effect of PKM2 is currently considered to be the interception of glucose metabolism and the metabolic pathway is transferred to the pentose phosphate pathway (PPP), the uronic acid pathway (UAP), the polyol pathway (PYP), etc. for the synthesis of the subsequent five-carbon ribose and non-essential amino acids. The TCA circle is backed up by fatty acid metabolism and amino acid metabolism, and its main purpose is to provide raw materials for the synthesis of non-essential amino acids, and the secondary purpose is to supply REDOX equivalents [43]. Although the classic glutathione replenishment pathway is well known. But there are more similar pathways in tumor cells which marked with blue text in the picture. PKM2 can be replenished by many amino acids such as alanine (Ala), cysteine (Cys), glycine (Gly), threonine (Thr), tryptophan (Try), etc. Although Ac-CoA mainly relies on fatty acid metabolism for supply, it can also be replenished by some amino acids such as leucine, isoleucine, tryptophan etc. The tricarboxylic acid cycle (TCA) serves as the focal point for the metabolism of the three major metabolic processes, the amino acid is also the most abundant in its form of replenishment. For example, a variety of amino acids such as aspartic acid, arginine, glutamic acid, glutamine, histidine, isoleucine, methionine, phenylalanine, proline, tyrosine, threonine, valine etc. can complement the eight intermediate metabolites in the tricarboxylic acid cycle (TCA). In the three major metabolic processes, the REDOX equivalents produced will converge in the mitochondria and eventually promote the oxidative phosphorylation (OXPHOS) of the electron transport chain, while protecting the mitochondrial function of the tumor cells, also providing the cells with a large amount of adenosine triphosphate (ATP) required for survival as well [44]. REDOX equivalents marked with purple text in the picture

Glucose is the main energy supply substance in normal tissues, under the condition of sufficient oxygen, glucose undergoes biological processes such as glycolysis, tricarboxylic acid cycle (TCA) and oxidative phosphorylation (OXPHOS) to completely decompose glucose into carbon dioxide and water, and when consumes oxygen, the cell itself is supplied with a large amount of ATP at the same time [13]. Warburg found that abnormal glucose metabolism is an important feature of tumor cells, that is, tumor cells under the conditions of oxygen enrichment, but with a less efficient aerobic glycolysis, and then proposed the famous “Warburg effect”, the tumor supplies energy through a low-efficiency ATP production process that uses glucose uptake for aerobic glycolysis [14]. Although the Warburg effect has been practiced for nearly a century, with the deepening of research on glucose metabolism in tumor cells, it has been found that although there is indeed high consumption of glucose in tumor tissues, there is a proportional difference between glucose consumption and ATP supply, the production of ATP is more than the corresponding amount of ATP produced by glucose aerobic glycolysis [15], therefore, after years of academic debate and continuous improvement of the second stage of Warburg effect, the speculation that most scholars can accept at this stage is that tumor cells divide glucose metabolism into three separate parts of glycolysis, tricarboxylic acid cycle (TCA), and oxidative phosphorylation (OXPHOS) [16], Dr. Warburg only explained the glycolysis part of glucose metabolism in tumor cells, namely aerobic glycolysis, which is produced by the effect is currently thought to be the interception of glucose metabolism rather than the shunt [17], the purpose of which is to switch glucose metabolites pathway of entering the tricarboxylic acid cycle, oxidizing the respiratory chain for complete oxidative decomposition into the pentose phosphate pathway, aldose acid pathway, polyol pathway etc. (Fig. 1 the section of glucose metabolism) for the synthesis of five-carbon ribose and non-essential amino acids [18], after that, provide the biomass needed for proliferation to the tumor cells, although a small amount of glucose can still follow the original Warburg effect to produce pyruvate by glycolysis, and the pyruvate shuttles to form Ac-CoA into the tricarboxylic acid cycle, which means both aerobic fermentation and the aerobic oxidation are parallel [19], but subsequent studies have found that a large amount of acetyl-CoA (Ac-CoA) entering the tricarboxylic acid cycle (TCA) is more likely to come from fatty acid oxidation (FAO), amino acid replenishment and gluconeogenesis pathway etc. [20], and the main purpose of the tricarboxylic acid cycle (TCA) is no longer supplies H^+^ and REDOX equivalents to the subsequent electron transport chain, but provides tumor cells such as Glutamine, Proline, Ornithine, Lysine, Methionine and other non-essential amino acids which tumor cells biosynthesis required for (Fig. 1 the section of amino acid complement) [21, 22]. In this knowledge framework, the tricarboxylic acid cycle (TCA) is more similar to the spinning gyro, which automatically balances the unstable factors, which also reveals why many experimenters simply inhibit the activity of a key enzyme in the tricarboxylic acid cycle (TCA) or remove some important intermediate metabolites, the tumor still survives [23, 24]. The reason is that when this gyro is given an unstable factor, the tumor cells can regulate other enzyme activities upstream and downstream of this unstable factor in the tricarboxylic acid cycle (TCA), and through the amino acid replenishment and balance the yield of each intermediate metabolites to reach the next dynamic equilibrium [25]. Therefore, it is suitable for tumor survival. After the glucose metabolism pathway of tumor cells turning to a pentose phosphate pathway, a uronic acid pathway, a polyol pathway, etc., also provides a large amount of REDOX equivalents such as NADPH, NADH and FADH2. Many researchers have realized that glutamine plays a crucial role in REDOX equivalent replenishment, and certain researchers have found that this model can produce a considerable amount of REDOX equivalents, however, few people are aware of the important role that essential and non-essential amino acids play in participating in REDOX equivalent replenishment. If only the REDOX equivalent production is considered to be a simple chemical equation, this new REDOX equivalent generation mode can produce about 70% of the TCA mode, which also reveals why the mitochondrial function still exists in the context of the traditional Warburg effect. These REDOX equivalents can be combined with fatty acid metabolism, amino acid replenishment, and reducing equivalents in the tricarboxylic acid cycle to form a REDOX equivalent pool [26, 27], while providing a carbon source for tumor cell proliferation, at the same time part of these REDOX equivalents will participate in the biosynthesis of tumor cells [28, 29], and the other part will bypass the TCA cycle and directly oxidize and phosphorylate to supply cells with ATP (Fig. 1 the section of purple part) [30]. Through fatty acid metabolism, protein metabolism and tricarboxylic acid cycle synergy to supplied hydrogen ions jointly maintain the membrane potential of the mitochondria and maintain the operation of the electron transport chain [21, 31]. The integrated metabolic mode of glucose, amino acid and fatty acid of the tumor cells protects the mitochondrial membrane function, ensures a large amount of ATP supply to the tumor cells [32], and it also provides a material basis for the proliferation of tumor cells [33, 34]. Therefore, when PKM2 is newly knocked out from the tumor and PKM1 is expressed, the mitochondrial respiration of the cancer cells is converted from aerobic glycolysis to mitochondrial respiration, and the tumor cell proliferation ability, invasion and metastasis ability are all decreased [35]. This conjecture is also referred to by some scholars as the “post–post-Warburg effect”, or maybe we can call it as “ZZ effect”, and this energy supply model is called “reprogramming of tumor cell energy metabolism (EMR)” [36, 37]. PKM2 combined with other key enzymes in glucose metabolism, such as glycosyl kinase (GK), pyruvate kinase (PK), pyruvate dehydrogenase kinase (PDK), lactate dehydrogenase (LDH), glucose transporters (GLUT), etc. [38]. These key enzymes work together to regulate tumor energy metabolism, and the effect is not the mode of the barrel short plate, that is, simply inhibiting the activity of an enzyme, although it can inhibit the metabolism of tumor ability in a short time course, it is quickly regulated by other key enzymes after a while and perform compensate [39]. The effect of inhibiting a single enzyme is called to anti-Matthew Effect would more closely. That is, the weakened enzyme has its activity compensation enhanced or compensated by the biological action of other enzymes, and the relatively strong enzyme weakens its biological function, thereby achieving a dynamic equilibrium relationship with the weakened enzyme [40–42] (Additional file 1: Table S1).

POST-Warburg effect, in glucose metabolism, tumor cells divide glucose metabolism into three separate parts of glycolysis, tricarboxylic acid cycle and oxidative phosphorylation (OXPHOS). The effect of PKM2 is currently considered to be the interception of glucose metabolism and the metabolic pathway is transferred to the pentose phosphate pathway (PPP), the uronic acid pathway (UAP), the polyol pathway (PYP), etc. for the synthesis of the subsequent five-carbon ribose and non-essential amino acids. The TCA circle is backed up by fatty acid metabolism and amino acid metabolism, and its main purpose is to provide raw materials for the synthesis of non-essential amino acids, and the secondary purpose is to supply REDOX equivalents [43]. Although the classic glutathione replenishment pathway is well known. But there are more similar pathways in tumor cells which marked with blue text in the picture. PKM2 can be replenished by many amino acids such as alanine (Ala), cysteine (Cys), glycine (Gly), threonine (Thr), tryptophan (Try), etc. Although Ac-CoA mainly relies on fatty acid metabolism for supply, it can also be replenished by some amino acids such as leucine, isoleucine, tryptophan etc. The tricarboxylic acid cycle (TCA) serves as the focal point for the metabolism of the three major metabolic processes, the amino acid is also the most abundant in its form of replenishment. For example, a variety of amino acids such as aspartic acid, arginine, glutamic acid, glutamine, histidine, isoleucine, methionine, phenylalanine, proline, tyrosine, threonine, valine etc. can complement the eight intermediate metabolites in the tricarboxylic acid cycle (TCA). In the three major metabolic processes, the REDOX equivalents produced will converge in the mitochondria and eventually promote the oxidative phosphorylation (OXPHOS) of the electron transport chain, while protecting the mitochondrial function of the tumor cells, also providing the cells with a large amount of adenosine triphosphate (ATP) required for survival as well [44]. REDOX equivalents marked with purple text in the picture (Additional file 2: Fig. S1).

**Fig. 2:**
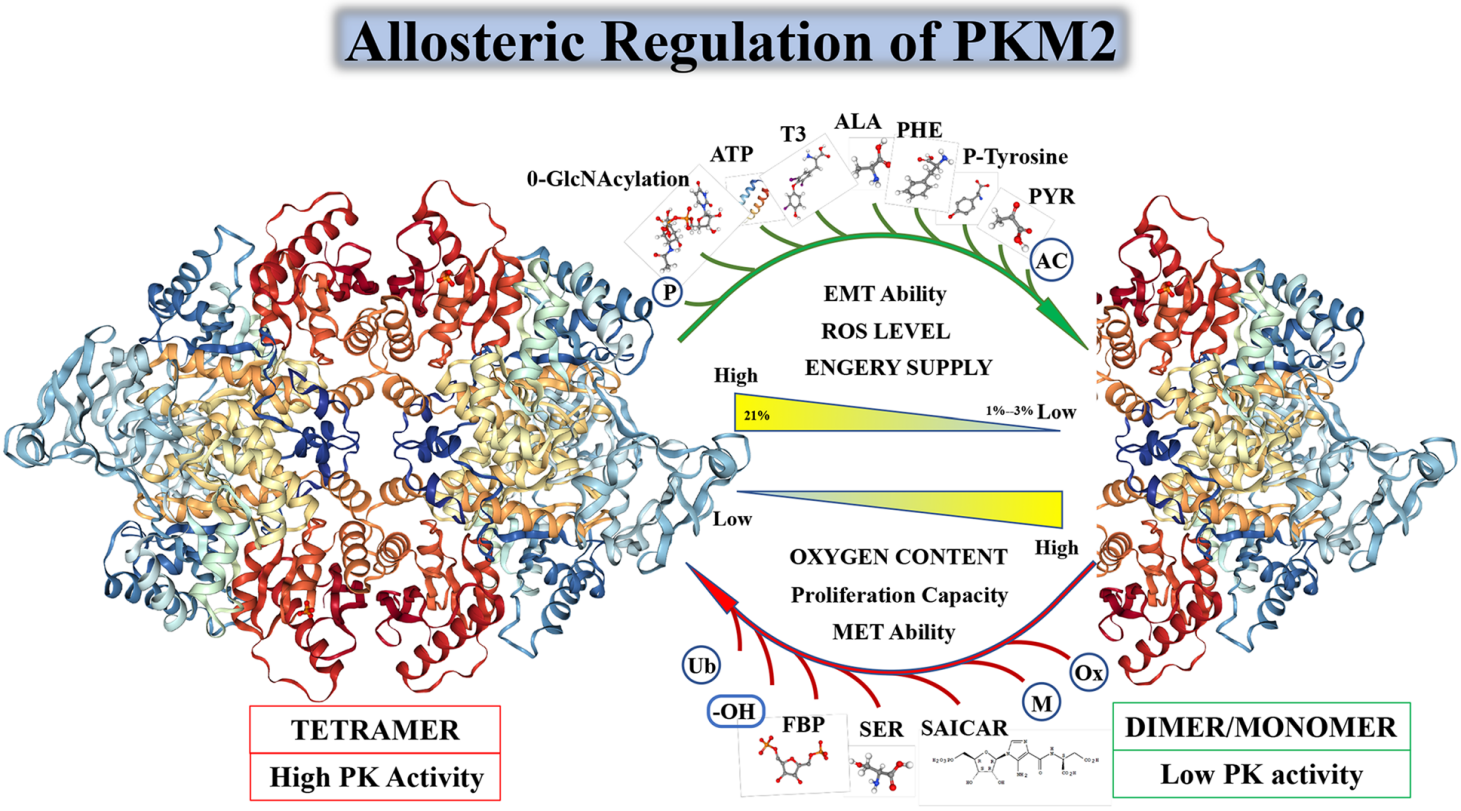
Relationship between PKM2 enzyme activity and spatial conformation. The transition between PKM2 dimers and tetramers is allosterically regulated by endogenous and exogenous activators and inhibitors. PKM2 has PK enzyme activity only when it serves as a tetramer. PKM2 is activated by the glycolytic intermediate products named fructose 1,6-bisphosphate (FBP). It can also be activated by the allosteric effects of serine and succinylaminoimidazolecarboxamide ribose-50 phosphate SDH succinate dehydrogenase (SAICAR) [54, 55]. The PK enzymatic activity of PKM2 can be inhibited by many endogenous inhibitors and cellular signaling events including 0-GlcNAcylation, pyruvate (PYR), P-tyrosine (P-TYR), phenylalanine (PHE), alanine (ALA), adenosine triphosphate (ATP), and thyroid hormone T3 [56–58]. In addition, due to the number of related molecules involved in PKM2’s post-translational modification (PTM), I will not list them in Fig. 2, but in the form of Tables 2 and 3 in the fourth part of this article “Interaction of PKM2 with other proteins”

Although PKM1, PKM2, PKL, and PKR all have a tetrameric form and pyruvate kinase activity, only PKM2 has both a dimeric form and a tetrameric form. In Fig. 2, we will show the characteristics of PKM2 in different states and its unique allosteric adjustment mode. When PKM2 is in a tetrameric state, it has a higher affinity with its substrate phosphoenolpyruvate (PEP), and a higher PK enzymatic activity, to catalyze the production of pyruvate by phosphoenolpyruvate (PEP). PKM2 also has a dimer state with low PK activity, and PKM2 is mainly present in the dimer state in the absence of environmental stress [45]. On the one hand, the dimeric PKM2 is closely related to the biosynthesis mentioned in the previous paragraph, on the other hand, the dimeric PKM2 can enter the nucleus as a transcription factor to activate the transcription of certain genes, and can also coordinate with other transcription factors to regulate the transcription of the gene, the specific mechanism of action will be described in the third part of this paper, “The non-glycolysis enzyme function of PKM2. Some scholars have suggested that the ratio of PKM2 dimer to tetramer state determines whether glucose metabolism in cells is involved in the biosynthesis of nucleic acids, proteins, amino acids or pyruvate into mitochondria to participate in energy metabolism [46]. The ratio between tetramer structure and dimer structure of PKM2 is regulated by environmental factors, oncogenes, tumor suppressor genes, and intermediate metabolites. When the proportion of dimers increase, it indicates that tumor cells accumulate mainly by glycolysis metabolites at this time, thus providing material preparation for tumor proliferation [47]. For example, 3-phosphoglycerate is an intermediate metabolite of glycolysis and a synthesis precursor of serine, glycine, cysteine, and sphingolipid. Phosphorylated dihydroxyacetone provides a backbone for phospholipid synthesis, and fructose 6-phosphate fructose and glyceraldehyde-3-phosphate synthesize ribonucleotides via the oxidized pentose phosphate pathway and the non-oxidized pentose phosphate pathway, respectively. Nucleotides, phospholipids, sphingolipids and amino acids are important components of cells and are essential for rapid cell proliferation. High levels of dimeric structure PKM2 lead to an increase in fructose 1,6-diphosphate, but when the concentration of fructose 1,6-diphosphate reaches a certain threshold, it indicates that tumor cell proliferation is blocked, and tumor cells face survival pressure [48], and low activity dimer structure PKM2 is reconverted to a highly active tetrameric structure, and tetramer allosteric activators have now been found to be: phenylalanine (Phe), alanine (Ala), thyroid hormone T3 (T3), p-tyrosine, growth signal, intracellular low reactive oxygen species (ROS) levels and fructose 1,6-diphosphate (FBP) can regulate the activity of PKM2 [49, 50]. In addition to the above substances, other substances that have a conditioned effect on the PKM2 tetramer include a similar regulation of glucose metabolism bypass products such as serine (SER), glycine (GLY), cysteine (CYS) etc. which promotes the re-polymerization of PKM2 into a tetrameric form therefore, catalyzes the conversion of glucose to lactic acid to generate energy until these allosteric modifiers fall below their threshold [51]. In addition, high levels of dimeric form of PKM2 aggregation can lead to an increase in upstream metabolites of the pyruvate kinase reaction in glycolysis, and is used for the synthesis of nucleotides, NADPH and phospholipids to ensure cell proliferation. Similarly, when tumor cells are exposed to stress or anti-chemotherapeutic drugs, such as increased expression of hypoxia inducible factor-1α (HIF-1α) in cells during hypoxia, both the transcriptional activity of PKM2 and the proportion of tetrameric PKM2 can be increased, similarly when cisplatin etc. using ROS as a killing chemotherapeutic agent that acts on tumor cells is used [52], PKM2 reforms into a tetrameric form, open the tricarboxylic acid cycle (TCA) and the electron transport chain to consume excess reactive oxygen species in the cell and protect the mitochondria from drug attack. In summary, amino acid, fatty acid, glucose intermediate and bypass metabolites can regulate PKM2 enzyme composition and enzyme activity [53] (Additional file 3: Fig. S2).

The transition between PKM2 dimers and tetramers is allosterically regulated by endogenous and exogenous activators and inhibitors. PKM2 has PK enzyme activity only when it serves as a tetramer. PKM2 is activated by the glycolytic intermediate products named fructose 1,6-bisphosphate (FBP). It can also be activated by the allosteric effects of serine and succinylaminoimidazolecarboxamide ribose-50 phosphate SDH succinate dehydrogenase (SAICAR) [54, 55]. The PK enzymatic activity of PKM2 can be inhibited by many endogenous inhibitors and cellular signaling events including 0-GlcNAcylation, pyruvate (PYR), P-tyrosine (P-TYR), phenylalanine (PHE), alanine (ALA), adenosine triphosphate (ATP), and thyroid hormone T3 [56–58]. In addition, due to the number of related molecules involved in PKM2’s post-translational modification (PTM), I will not list them in Fig. 2, but in the form of Tables 2 and 3 in the fourth part of this article “Interaction of PKM2 with other proteins” (Additional file 4: Fig. S3).

**Fig. 3:**
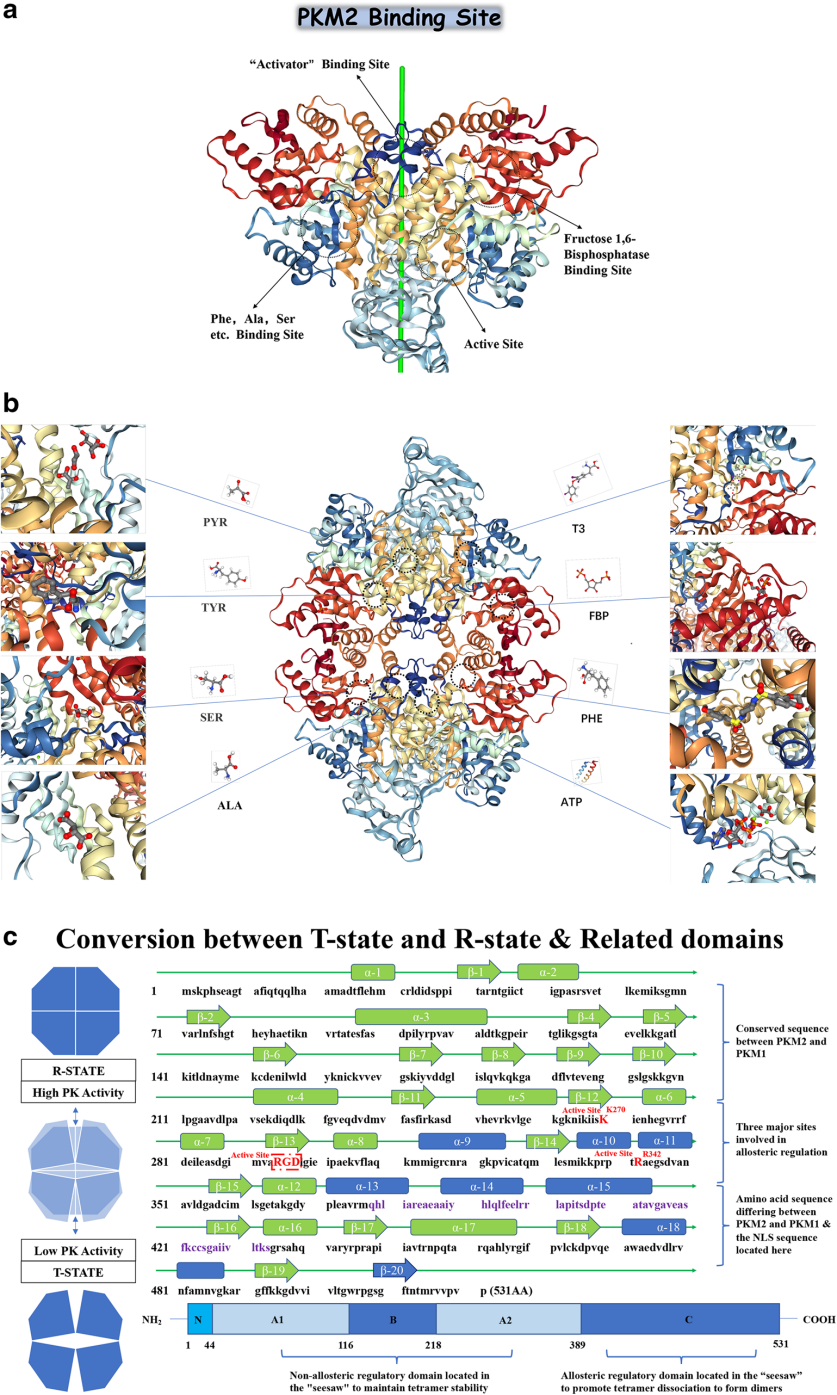
The Specific Site of PKM2 Allosteric Regulation and The Amino Acid Sequence of PKM2. It is now accepted that the PKM2 in the tetrameric state has an allosteric regulatory domain within its spatial structure, forming a pattern similar to the seesaw, when some allosteric regulators are inserted into the spatial structure involved in PKM2 allosteric regulation. After the domain (**a**), the tetramer PKM2 can be transferred from a compact state (R-state) to a loose state (T-state) and finally disassembled into a dimeric form [59]. When these allosteric regulators bind to PKM2, they will change the spatial conformation of PKM2, and affect the electrostatic force inside the molecule, and then affect the transition state of PKM2. The allosteric form a stable and compact PKM2 R-state to form a tetramer and perform PK enzyme activity. After allosteric adjustment the PKM2 forms a loose and unstable T-state, and eventually breaks the linked fragment in the tetramer to form a PKM2 dimer form with lower PK enzymatic activity. When PKM2 is allosteric to form a dimer, it will expose the active region inside the molecule, although the PK enzyme activity is low, it has protein activation activity [60]. In **b** we specifically list the each participating allosteric regulates the binding site of the small molecule and the binding site of the activator in the PKM2 protein spatial structure [61]. In Fig. 3c we simply describe the seesaw structure of PKM2 and specifically identify the specific spatial domains that participate in the seesaw pattern: α-9 and 10 and 11 and 13 and 14 and 15 and 18 and β-20 which marked with blue text in **c**. The residues at the active site which was mentioned in **a** are highlighted by red box in **c**. Residue Arg342 and Residue Lys342, which is responsible for active site “RGD” stabilization is colored in red. There is one point to be noted here: whether PKM2 has protein kinase activity or not, there is a negative attitude, however in some of the researchers’ experiments described later, phosphorylation was indeed found [62]

It is now accepted that the PKM2 in the tetrameric state has an allosteric regulatory domain within its spatial structure, forming a pattern similar to the seesaw, when some allosteric regulators are inserted into the spatial structure involved in PKM2 allosteric regulation. After the domain (Fig. 3a), the tetramer PKM2 can be transferred from a compact state (R-state) to a loose state (T-state) and finally disassembled into a dimeric form [59]. When these allosteric regulators bind to PKM2, they will change the spatial conformation of PKM2, and affect the electrostatic force inside the molecule, and then affect the transition state of PKM2. The allosteric form a stable and compact PKM2 R-state to form a tetramer and perform PK enzyme activity. After allosteric adjustment the PKM2 forms a loose and unstable T-state, and eventually breaks the linked fragment in the tetramer to form a PKM2 dimer form with lower PK enzymatic activity. When PKM2 is allosteric to form a dimer, it will expose the active region inside the molecule, although the PK enzyme activity is low, it has protein activation activity [60]. In Fig. 3b we specifically list the each participating allosteric regulates the binding site of the small molecule and the binding site of the activator in the PKM2 protein spatial structure [61]. In Fig. 3c we simply describe the seesaw structure of PKM2 and specifically identify the specific spatial domains that participate in the seesaw pattern: α-9 and 10 and 11 and 13 and 14 and 15 and 18 and β-20 which marked with blue text in Fig. 3c. The residues at the active site which was mentioned in Fig. 3a are highlighted by red box in Fig. 3c. Residue Arg342 and Residue Lys342, which is responsible for active site “RGD” stabilization is colored in red. There is one point to be noted here: whether PKM2 has protein kinase activity or not, there is a negative attitude, however in some of the researchers’ experiments described later, phosphorylation was indeed found [62].

## The splicing of PMK2

**Fig. 4:**
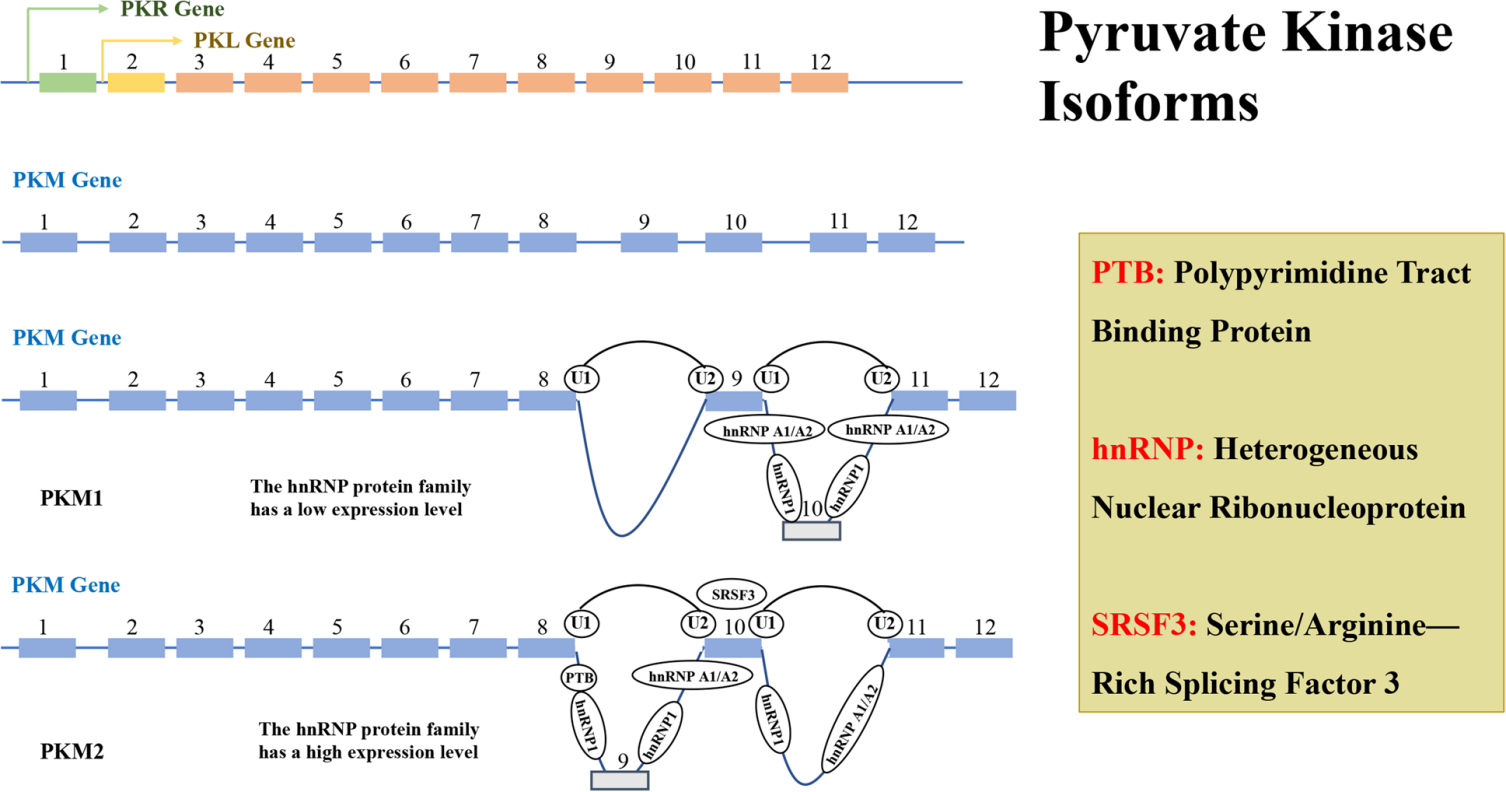
The Splicing of PMK2. The genes encoding pyruvate kinase can be divided into two types: PKLR and PKM. PKLR binds to the coding gene through a tissue-specific promoter, encoding two subtypes of PKL and PKR (green for PKR and yellow for PKL). PKM encodes PKM1 and PKM2 subtypes by alternative splicing of mutually exclusive exon 9 and 10, and a high expression level of PTB, hnRNP 1 & hnRNP A1/A2 are required for during the cleavage process of exon 9 of PKM2, while the cleavage process of exon 10 of PKM1 is not required. Transcription factor SRSF3 also plays an important role in the conversion of PKM2 and PKM1. Each pyruvate kinase subtype has a different tissue expression pattern

**Fig. 5:**
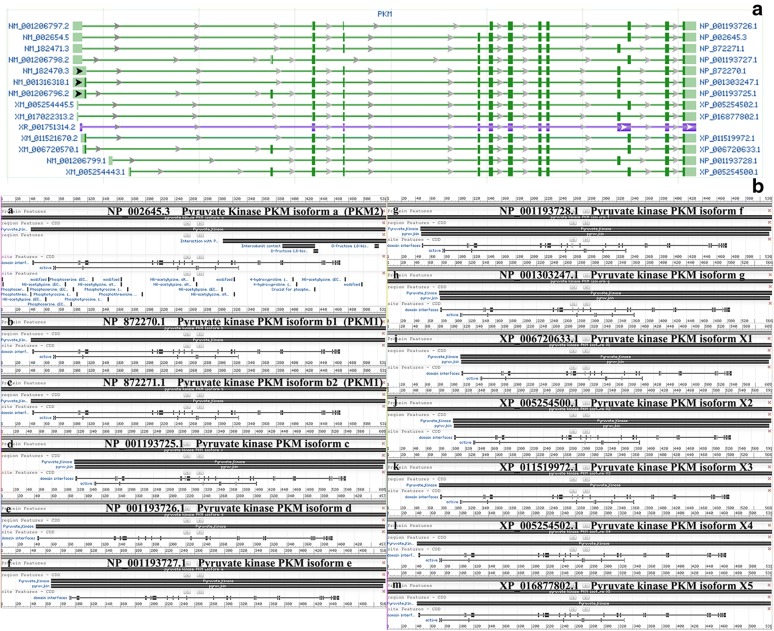
Expression Patterns of The Transcript and The Protein Subtypes of PKM. **a** That there are 14 different subtype sequences in the PKM gene transcript, and there are some differences between them, among which the PKM1 (No. 3 and No. 5) and PKM2 (No. 2) subtypes are more compared, **b** the coding sequence NO. 12 expresses PKM1 and the coding sequence expresses NO. 13 PKM2. There are only 23 amino acid residues between the two protein sequences. And there are few studies on other PKM protein subtypes, and the functions are not sure yet

There are four PK subtypes encoded by two genes in mammals which was shown in Fig. 4. The PK enzyme is encoded by two genes, PKLR and PKM, which PKL gene encodes PKL, and is expressed in the liver and pancreas, intestine, and kidney, while PKR is mostly expressed in red blood cells [63]. PKM1 and PKM2 encoded by PKM gene have the same length of gene coding and alternative mutually exclusive exons, could encode 56 amino acid residues, and the regions differ in the splicing difference at the 22nd position [64, 65]. The PKM subtypes perform the same catalytic function. However, in view of the fact that PKM1 is a tetrameric enzyme with sustained activity, there is a difference of 22 amino acids when compared with PKM2, and the mRNA generated by transcription of PKM under the action of cleavage factor, can form PKM1 containing exon 9 or PKM2 containing of exon 10 [66]. In Fig. 5 and Table 1, we could see the splicing factors of PKM gene include: hnRNPL (PTB), hnRNPAI, hnRNPA2 three heterogeneous riboproteins, which release exon 10 by binding exon 9, and promote PKM2 expression while inhibit PKM1 expression, NEK2 (never in mitosis (NIMA)-related kinase 2) can promote the release of exon 10 by binding to hnRNPAI/A2, further increase the expression of PKM2 [67–69]. Intensive studies have shown that under the regulation of C-MYC, three hnRNPs (heterogeneous ribonucleoproteins): hnRNPL (PTB), hnRNPA1, hnRNPA2, bind to the intron sequence between exon9 and exon10, inhibits the cleavage of exon9, while serine/arginine-rich protein-specific kinase (SRSF-3) combines with the exon10 sequence to facilitate exon10 cleavage, thus completing the conversion of PKM1 to PKM2 [70, 71]. This difference is located in the spatial groove of PKM2 combined with fructose-1,6-diphosphate (FBP), allowing FBP to bind to PKM2 to deform the latter into an active tetramer. HIF-1α induces the PI3 K-AKT-mTOR signaling pathway and also regulates PKM2 by down-regulating C-MYC expression and up-regulating hnRNPs [72, 73]. NF-κB can mediate transcriptional upregulation of the PKM gene [74]. When epidermal growth factor (EGF) acts and activates its receptor (EGFR), EGFR can also induces NF-κB activation following inflammatory and cytokine stimulation, in which polyubiquitination of IKK and phosphorylation of TAK1 plays a crucial role this process. Activation of EGFR mediates PLCγ1-dependent PKCε activation, resulting in PKCε monoubiquitination of Lys321 by RINCK1 ubiquitin ligase. Monoubiquitinated PKCε interacts with the NEMO zinc finger domain and recruits the cytoplasmic IKK complex to the plasma membrane, where PKCε phosphorylates IKKβ and activates IKKβ at Ser177 [75]. Activated RelA interacts with hypoxia-inducible factor 1 alpha (HIF1α), ultimately binds RelA to the PKM gene promoter and activates PKM transcription [76]. In turn, PTB is also up-regulated by EGFR activation, and the PKM pre-mRNA is spliced into PKM2 mRNA to up-regulate PKM2 expression. These results indicate that both PKM and precursor mRNA are produced by transcription of the PKM gene, but the transition of PKM1 to PKM2 expression is a coordinated regulation of PTB-dependent splicing [77]. A total of 14 transcripts and 12 protein subtypes of the PKM gene are recorded in the NCBI and UCSC databases, see Table 1 for details. For broader regions represent the exons and the narrower regions represent introns. The dark regions represent the sequence between the translation initiation codon and the stop codon, and the light regions represent the 57 UTR and 37 UTR regions [78] (AA: amino acid residues).

**Table 1:** Subtype classification of pyruvate kinase in mammals

Serial number	NCBI reference sequence	Pyruvate kinase PKM isoform [Homo sapiens]	Portion
1	NP_001193726.1	Pyruvate kinase PKM isoform d	457AA
2	NP_002645.3	Pyruvate Kinase PKM isoform a	Pyruvate kinase 2 (PKM 2) 531AA
3	NP_872271.1	Pyruvate kinase PKM isoform b2	Pyruvate kinase 1 (PKM 1) 531AA
4	NP_001193727.1	Pyruvate kinase PKM isoform e	531AA
5	NP_872270.1	Pyruvate kinase PKM isoform b1	Pyruvate kinase 1 (PKM 1) 531AA
6	NP_001303247.1	Pyruvate kinase PKM isoform g	566AA
7	NP_001193725.1	Pyruvate kinase PKM isoform c	605AA
8	XP_005254502.1	Pyruvate kinase PKM isoform X4	
9	XP_016877802.1	Pyruvate kinase PKM isoform X5	
10	NONE		
11	XP_011519972.1	Pyruvate kinase PKM isoform X3	
12	XP_006720633.1	Pyruvate kinase PKM isoform X1	
13	NP_001193728.1	Pyruvate kinase PKM isoform f	536AA
14	XP_005254500.1	Pyruvate kinase PKM isoform X2	

In addition to the different cleavage patterns, more and more studies have shown that the expression of PKM2 is also closely related to MicroRNAs (miR/miRNA), Long non-coding RNAs (LncR/LncRNA), etc., in non-coding RNA families, among which miRNAs are a class of short-chain non-coding RNAs, and are bound to the seed region at the ‘-UTR end of mRNAs and conduct functions, therefore affect protein synthesis and folding [79]. Some scholars have found that there are two binding sites at the 3′ UTR end of miRNA-326 and PKM2, and miRNA-326 can inhibit the expression of PKM2 in glioma cells [80]. In intestinal cancer cells, miRNA-let-7a inhibits the proliferation, invasion and migration of intestinal cancer by down-regulating the expression of PKM2 [81–83]. Some microRNAs have organ specification, such as miRNA-122 [84], miRNA-124 [85], miRNA-133-3p [86], miRNA-137 [84], miRNA-20 [68] [4, 87] and miRNA-3662 [88] etc., which regulate the expression of PKM subunits by directly targeting polypyrimidine bundle binding protein 1 (PTBP1), while polypyrimidine bundle binding protein 1 (PTBP1) is a splice that regulates PKM2 dominant expression. Similar to: miRNA-29b [89], miRNA-99a [90], miRNA-133b [91], miRNA-145 [92], miRNA-148a [93], miRNA-152 [93, 94], miRNA-290 [95], miRNA-326 [96], miRNA-338-3P [97], miRNA-340 [98], miRNA-369 [99], miRNA-371 [95], miR-379 [100], miRNA-675 [101], miRNA-4417 etc. [102]. While these miRNAs are targeted to the splicing factors PTBP1, hnRNPA1, and hnRNPA2, [103] the expression of PKM mRNA is shifted from PKM1 to PKM2, and the expression of PKM2 is increased [104]. In addition to the important total use of miRNAs in epigenetics, there is also a long-chain non-coding RNA called LncRNA in cells. Although these RNAs are difficult to directly regulate the mRNA of PKM2, they can regulate miRNAs binding of PKM2, in turn to regulate the expression of PKM2. For example, miRNA-675 can form a ceRNA model with LncRNA-H19 [105], which in turn affects PKM2 expression. The same regulation exists between LncRNA-MEG3 and miRNA-122 [106], LncRNA-MIF and miRNA-586 [107], LncRNA-CASC2c and miRNA-101 [97]. The double-mutant P53 can regulate LncRNA CUDR and down-regulate PKM2 to inhibit tumor growth [108]. In addition to the regulation of tumor suppressor genes, these long-chain non-coding RNAs can also influence intracellular signaling pathways, for example, LncRNA-Ftx [109], LncRNA-SchLAH [110], LncRNA-ROR [111], LncRNA-DACOR1 [112] can mediate PTEN signaling pathway in cells. The Pi3k/AKT/mTOR signaling pathway has an effect that produces different biological effects [72, 113].

The genes encoding pyruvate kinase can be divided into two types: PKLR and PKM. PKLR binds to the coding gene through a tissue-specific promoter, encoding two subtypes of PKL and PKR (green for PKR and yellow for PKL). PKM encodes PKM1 and PKM2 subtypes by alternative splicing of mutually exclusive exon 9 and 10, and a high expression level of PTB, hnRNP 1 and hnRNP A1/A2 are required for during the cleavage process of exon 9 of PKM2, while the cleavage process of exon 10 of PKM1 is not required. Transcription factor SRSF3 also plays an important role in the conversion of PKM2 and PKM1. Each pyruvate kinase subtype has a different tissue expression pattern (Additional file 5: Fig. S4).

Figure 5a shows that there are 14 different subtype sequences in the PKM gene transcript, and there are some differences between them, among which the PKM1 (No. 3 and No. 5) and PKM2 (No. 2) subtypes are more compared, Fig. 5b shows the coding sequence NO. 12 expresses PKM1 and the coding sequence expresses NO. 13 PKM2. There are only 23 amino acid residues between the two protein sequences. And there are few studies on other PKM protein subtypes, and the functions are not sure yet.

## Non-glycolysis enzyme function of PKM2

**Fig. 6:**
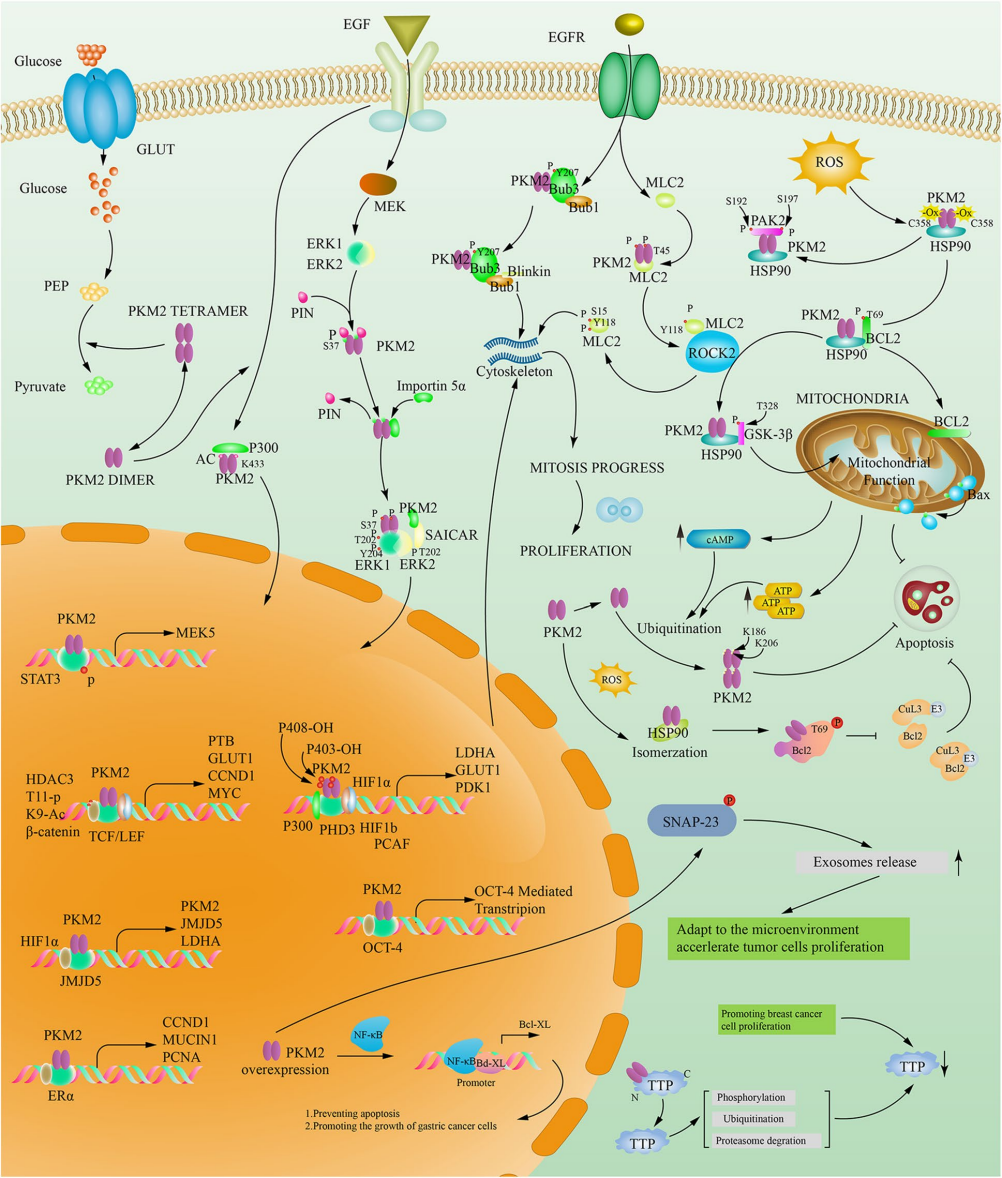
PKM2 Interacting Protein. PKM2 can not only exert the activity of PK enzyme in the form of tetramer, but also can enter the nucleus as a transcription factor to mediate the transcription of other genes when convert to dimer, can also regulate each other in the cytoplasm and other proteins. It has an impact on many different biological effects

By learning of that, it could be found PKM2 not only plays an important role in cytosolic glucose metabolism, but also can transfer from the cytoplasm to the nucleus rely on its c-terminal nuclear localization signal in interleukin-3, growth hormone inhibitor analogue TIT-232, peroxide, epidermal growth factor (EGFR), ultraviolet radiation and other factors, and in the form of dimers to play a role in protein kinase activity in the nucleus of a variety of transcription factors and thus affect a variety of signaling pathways to promote tumor development [114, 115]. Recent studies have found that PKM2-specific exon 10 can recruit extracellular signal-regulated kinase 2 (ERK2) and bind to the Iso429/Leu431 region of PKM2, mediate Ser37 site phosphorylation on PKM2, and recruit PIN1 to form PRKM2/PIN1 complex, if the Ser37 site is mutated to other amino acids, although PKM2 can still form tetramers in the cytoplasm, PKM2 cannot enter the nucleus [116], which suggests that the complex is an important transporter that mediates PKM2 entry into the nucleus, all these results suggest that PKM2 can play a role in regulating transcription and post-translational modification, and these effects depend on the interaction between PKM2 and ERK1/2, PIN1 and Importin 5α [117]. Moreover, if PKM2 exists in the form of a tetramer, the Arg399 site of nuclear localization sequence (NLS) in one side of the PKM2 monomer in its symmetrical structure can form a stable charge–charge interaction with Glu418 and Glu396 of the opposite mirror PKM2 monomer (the other two is located in the tetramer PKM2), which also maintains the spatial conformation of the PKM2 tetramer to some extent. Only when the spatial structure of PKM2 changes, such as PKM2 in the dimeric form, fully exposes the NLS of PKM2 which buried in the 3-dimensional space, through interacting with Importin 5α and NLS to change the electrostatic attraction inside the PKM2 protein molecule after binding the PIN1 molecule, in turn, the dimeric structure of PKM2 is maintained [118]. PKM2 also transactivates SLC2A1, LDHA, PDK1, HK1, and VEGFA gene expression via hypoxia inducible factor-1α (HIF-1α) transcription factor [56, 119]. In addition, PKM2 up-regulates gene expression through hypoxia inducible factor-1α (HIF-1α), β-catenin (β-cat), insulin, signal transducers and activators of transcription 3 (STAT3) and other transcription factors to promote cell growth and proliferation [74, 120, 121]. PKM2 can also act as a protein kinase, which forms a complex with β-catenin and binds to the CCND1 promoter region to phosphorylate histone H3, and phosphorylated histone H3 is separated from histone deacetylase 3, which will make the 9th tyrosine of histone H3 acetylation [122], then promotes the expression of an important cytokine of cell proliferation namely CycinD1, and then regulates the cell cycle. PKM2 can also affect the target gene C-MYC by affecting β-catenin, and C-MYC can also be used to promote PKM2 gene transcription and hnRNA cleavage in turn [123]. In addition to regulating the expression of CycinD1, which affects the transformation of G1-S stage, PKM2 also regulates the filamentous manner, which regulates the tyrosine at position 207 of the mitotic protein Bub3, promotes the formation of the Bub3-Bubl complex, and the Bub3-Bubl complex further act on the exocentric protein Blinkin to promote its binding to the ligand Bub and thus precisely regulates chromosome segregation and cell proliferation [124]. Studies have shown that under various conditions, the MER/ERK pathway is activated, phosphorylating the serine at position 37 on PKM2, which could help PKM2 enter the nucleus [125]. C-src can also be activated by EGFR activation, and C-src phosphorylates tyrosine at position 333 on β-catenin, so that PKM2 can bind to β-catenin/TCF/LEF after entering nucleation, thereby activates the expression of β-catenin downstream gene, such as CCND1 and MYC promote cell proliferation, but also activate PTB, LDHA, GLUT1. PTB can again activate the transcription of PKM2 to form a chain reaction [76]. LDHA and GLUT1 can regulate glucose uptake and tumor cell glycolysis, thereby regulate glucose metabolism. It was confirmed by research that PKM2 in the nucleus can phosphorylate tyrosine at 705th position of signal transduction factor and transcriptional activator 3, further promoting: the transcription of mitogen activated protein kinase kinase 5 (MAPKK5/MEK5), and MEK5 promotes the growth of tumor cells [126]. Li et al’s study confirmed that PKM2 is resistant to gemcitabine in patients with intestinal cancer by phosphorylating transcriptional activator factor 3. It has also been reported in the literature that PKM2 can make the serine at position 202/203 of protein kinase B substrate l (AKT1S1), promote its binding to 14-3-3 to activate mammalian rapamycin target protein sensitive complex 1 (mTORC l) signaling pathway to promote tumor growth [127]. PKM2 can directly phosphorylate extracellular signal-regulated kinase 1/2 (ERK l/2) to promote tumor growth [55]. Studies have shown that PKM2 can directly bind to the active domain of hypoxia inducible factor-1α (HIF-1α) to promote the activation of downstream vascular endothelial growth factor (VEGF) expression [128]. NAD-dependent deacetylase sirtuin6 (SIRT 6) could deacetylate the 433th lysine of PKM2 in the nucleus to cause it to transfer out of the nucleus and inhibit tumor growth. In summary, PKM2 in the nucleus is associated with cell proliferation [129]. Tables 2, 3 and Fig. 6 summarizes the specific sites, source literature, and biological effects of the various roles mentioned above.

**Table 2:** PKM2 interacting protein & Interaction site & Biological Function & References

PKM2 interacting protein				
	Protein	Site	Biological function	References
1	A-Raf	Phosphorylation modification, unknown site	Regulate the composition ratio between PKM2 dimer and tetramer, and then regulate the glucose metabolism of tumor cells	Pyruvate kinase type M2: a key regulator of the metabolic budget system in tumor cells
2	AKT1S1	PKM2 phosphorylates AKT1S1 at serine202 and serine203 (S202/203)	Activation of ATP-dependent mTOR signaling pathways	Pyruvate Kinase M2 Activates mTORC1 by Phosphorylating AKT1S1
3	BCL2	PKM2 phosphorylates Bcl2 threonine (T-69) (HSP90 stabilizes PKM2-BCL2 complex)	The phosphorylation prevents the binding of Cul3-based E3 ligase to Bcl2 and subsequent degradation of Bcl2, thereby inhibiting oxidative stress-induced apoptosis	Mitochondrial PKM2 regulates oxidative stress-induced apoptosis by stabilizing Bcl2
4	BCR-ABL	Phosphorylation modification, unknown site	Fusion of breakpoint cluster region and ABL1, disruption of the formation of the tetrameric form of PKM2	Association of the src gene product of Rous sarcoma virus with cytoskeletal structures of chicken embryo fibroblasts.
5	β-catenin	The k433 site of PKM2 is a key site for binding to y333 phosphorylated β-catenin	PKM2-β-catenin (Y333p) can promote the proliferation and malignant transformation of tumor cells	Nuclear PKM2 regulates β-catenin transactivation upon EGFR activation
6	CD44	Phosphorylation modification, unknown site	PKM2 binds to CD44 to inhibit phosphorylation of serine at a certain position in PKM2, thereby promoting aerobic glycolysis and promoting glucose metabolism to biosynthesis	Tyrosine phosphorylation inhibits PKM2 to promote the Warburg effect and tumor growth
7	CARM1	Methylated PKM2 exon 10, the specific location is unknown	PKM2 methylated by Carm1 can promote aerobic glycolysis and malignant transformation of tumors	PKM2 methylation by CARM1 activates aerobic glycolysis to promote tumorigenesis
8	ERK1/2	After binding to SAICAR, PKM2 phosphorylates ERK1 at t202, y204 sites and ERK2 to t202 siteAnd ERK2 phosphorylates PKM2 at s37 site	Erk and PKM2 together form a positive feedback activation loop that activates the ERK/MAPK pathway Phosphorylated PKM2 can increase its nuclear localization and promote cell proliferation	SAICAR induces protein kinase activity of PKM2 that is necessary for sustained proliferative signaling of cancer cells
9	ETV6–NTRK3	Phosphorylation modification, unknown site	Fusion of Est variant 6 and neurotrophic tyrosine kinase receptor, disruption of the formation of the tetrameric form of PKM2	Modulation of type M2 pyruvate kinase activity by the human papillomavirus type 16 E7 oncoprotein
10	HERC-1	Binding to PKM2 at AA 406–531	GTP producer for guanine nucleotide exchange factor RLD1	Interaction between HERC1 and M2-type pyruvate kinase
11	HIF-1a	Binding to PKM2	Augmentation of the trans-activating activity of HIF-1a	Pyruvate kinase M2 is a PHD3-stimulated coactivator for hypoxia-inducible factor 1
12	HCV NS5B	Binding to PKM2	Indications for a role of PKM2 in HCV RNA synthesis	Hepatitis C virus RNA functionally sequesters miR-122
13	HPV-16 E7	Binding to PKM2	Dimerization and inhibition of PKM2	Effects of the human papilloma virus HPV-16 E7 oncoprotein on glycolysis and glutami- nolysis: role of pyruvate kinase type M2 and the glycolytic-enzyme complex.
14	HSC70	Acetylation of Lys305 site for PKM2	By inhibiting the biological effects of PKM2 by acetylation of the PKM2 Lys305 site, it mediates the binding of PKM2 to HSC70 and attenuates the degradation of PKM2 via the lysosomal pathway	Acetylation targets the M2 isoform of pyruvate kinase for degradation through chaperone—mediated autophagy and promotes tumor growth
15	HSP70	Acetylation modification, unknown site	HSP70 can acetylate PKM2, which mediates intracellular localization of lysosomes, thereby maintaining intracellular homeostasis	Proviral insertion in murine lymphomas 2 (PIM2)oncogene Dual roles of PKM2 in cancer metabolism
16	H3	PKM2 can phosphorylate histone H3 T11	Phosphorylated histones can promote the G1-S phase transition of tumor cells, phosphorylation of stat3 can promote the production of cyclinD, phosphorylation of MLC2 can enhance the activity of MAPK pathway, and phosphorylated Bub3 can enhance the activity of EGFR pathway	Pyruvate kinase M2 at a glance
17	STAT3	PKM2 can phosphorylate stat3 Y705		
18	MLC2	PKM2 can phosphorylate MLC2 Y118		
19	Bub3	PKM2 can phosphorylate Bub3 Y27 site		
20	FLT3	Phosphorylation modification, unknown site	Fms-related tyrosine kinase, internal tandem duplication (ITD) mutant, disruption of the formation of the tetrameric form of PKM2	Association of the src gene product of Rous sarcoma virus with cytoskeletal structures of chicken embryo fibroblasts.
21	FGFR1	FGFR1 can phosphorylates PKM2 atTyr 83, Tyr105, Tyr148, Tyr175, Tyr370, Tyr390 site	Inhibits the biological activity of PKM2 which coule regulate the glucose metabolism in tumor cells	TRIM35 Interacts with pyruvate kinase isoform M2 to suppress the Warburg effect and tumorigenicity in hepatocellular carcinoma
22	JAK2	Phosphorylation modification, unknown site	Disruption of the formation of the tetrameric form of PKM2	Association of the src gene product of Rous sarcoma virus with cytoskeletal structures of chicken embryo fibroblasts.
23	JNK1	JNK1 phosphorylates PKM2 at Thr365 site	Inhibits the biological activity of PKM2 which coule regulate the glucose metabolism in tumor cells, and the nuclear transfer of PKM2 is inhibited, which in turn inhibits the biological role of PKM2 in the nucleus	PARP14 promotes the Warburg effect in hepatocellular carcinoma by inhibiting JNK1-dependent PKM2 phosphorylation and activation
24	GSK-3β	GSK-3β phosphorylation of PKM2 Thr-328 (HSP90 stable PKM2-GSK3β complex)	Thr-328 phosphorylation is essential for maintaining PKM2 stability and its biological function in regulating glycolysis, mitochondrial respiration, proliferation and apoptosis	HSP90 promotes cell glycolysis, proliferation and inhibits apoptosis by regulating PKM2 abundance via Thr-328 phosphorylation in hepatocellular carcinoma
25	P53	Phosphorylation modification, unknown site	PKM2-P53 can promote the proliferation and malignant transformation of tumor cells	Dual roles of PKM2 in cancer metabolism
26	p300	PKM2 could be acetylated by p300 at K433 site	PKM2 K433 acetylation converts cell proliferation and cytoplasmic metabolic kinase to nuclear protein kinase activity	Mitogenic and oncogenic stimulation of K433 acetylation promotes PKM2 protein kinase activity and nuclear localization
27	PAK2	PKM2 directly phosphorylates PAK2 (HSP90-stabilized PKM2-PAK2 complex) on Ser20, Ser141 (phosphorylated but weakly acting) and ser192/197 (action site)	Phosphorylation of serine 192/197 mediated by pkm2 in PDAC cells is critical for maintaining PAK2 levels phosphorylation of ser192/197 promotes the association of HSP90 with PAK2, thereby preventing ubiquitin and protease degradation of PAK2	Pyruvate kinase M2 promotes pancreatic ductal adenocarcinoma invasion and metastasis through phosphorylation and stabilization of PAK2 protein
28	PANK-4	Binding to PKM2	Reduction of the activity of the tetrameric form	Dual roles of PKM2 in cancer metabolism
29	Parkin	Parkin promotes ubiquitination of Lys186 and Lys206 sites in PKM2	Parkin inhibits the biological activity of PKM2 and regulates glucose metabolism by promoting ubiquitination of Lys186 and Lys206 sites of PKM2	Parkin regulates the activity of pyruvate kinase M2
30	PCAF	Acetylates modification, unknown site	Reduction of the activity of the tetrameric form	Dual roles of PKM2 in cancer metabolism
31	PHD3	PDH3 can hydroxylate the Pro403 and Pro408 sites of PKM2	PKM2 binds to PDH3, and modified PKM2 is more susceptible to HIF-1α binding and forms an activation loop that promotes anaerobic glycolysis and metabolic recombination	Pyruvate kinase M2 is a PHD3—stimulated coactivator for hypoxia—inducible factor 1
32	PKC ♁	Phosphorylation modification, unknown site	Hypothesis: regulation of stability or degradation of M2-PK	Dual roles of PKM2 in cancer metabolism
33	PIAS3	Binding to PKM2 at AA 1–348	Sumoylation of PKM2 and nuclear translocation of PKM2	The SUMO-E3 ligase PIAS3 targets pyruvate kinase M2
34	PML	Binding to PKM2	Reduction of the activity of the tetrameric form	Modulation of M2-type pyruvate kinase activity by the cytoplasmic PML tumor suppressor protein
35	PIM2	PIM2 can phosphorylate PKM2 at Thr454 site	PIM2 phosphorylates the PKM2 Thr454 site, mediates PKM2-dependent anaerobic glycolysis, and maintains mitochondrial function in tumor cells	Proviral insertion in murine lymphomas 2 (PIM2)oncogene phosphorylates pyruvate kinase M2 (PKM2) and promotes glycolysisin cancer cells
36	PTP1B	PTP1B phosphorylates the Tyr105 and Tyr148 site of PKM2	PTP1B inhibits the biological activity of PKM2 by phosphorylating the Tyr105 and Tyr148 site of PKM2	Protein tyrosine phosphatase 1B regulates pyruvate kinase M2 tyrosine phosphorylation
37	PRMT4	PRMT4 methylates specifically the dimeric form of PKM2 at Arg445/447/455 residues in the C domain	Allosteric activators inhibit PKM2 tetramerization form thought PKM2 methylation	Posttranslational modifications of pyruvate kinase M2: tweaks that benefit cancer
38	MG	MG can glycosylate the Arg399 site of PKM2	MG can glycosylate the Arg399 site of PKM2, the result of which can change the spatial configuration of PKM2	Molecular association of glucose-6-phosphate isomerase and pyruvate kinase M2 with glyceraldehyde-3-phosphate dehy-drogenase in cancer cell
39	Oct No. 4	Binding to PKM2 at AA 307–531	Augmentation of the trans-activating activity of Oct 4	Pyruvate kinase isozyme type M2 (PKM2) interacts and cooperates with Oct-4 in regulating transcription.
40	Opa	Binding to PKM2 at AA 367–531	Outer membrane proteins involved in gonococcal adhesion to and invasion of human epithelial cells creation of a microenvironment of high pyruvate concentration	Posttranslational modifications of pyruvate kinase M2: tweaks that benefit cancer
41	O_GlcNAcylation	O-GlcNAcylation can block the Thr 405 and Ser 406 sites of PKM2	Decreased the stability of the tetrameric form of PKM2 to enhance aerobic glycolysis (Warburg effect)	O-GlcNAcylation destabilizes the active tetrameric PKM2 to promote the Warburg effect
42	SAICAR	Mutation of the G415 site of PKM2 to R will not bind to SAICAR	PKM2 binds to SAICAR, and mutations at the PKM2 G415 site prevent PKM2 from binding to SAICAR	SAICAR induces protein kinase activity of PKM2 that is necessary for sustained proliferative signaling of cancer cells
43	SIRT-6	Deacetylation modification, the site is unknown	SIRT6 mediates the deacetylation of PKM2, and the results mediate the nuclear localization of PKM2	SIRT6 deacetylates PKM2 to suppress its nuclear localization and oncogenic functions
44	SOCS-3	Binding to PKM2	Reduction of ATP production and influence of dendritic cell immune response	Posttranslational modifications of pyruvate kinase M2: tweaks that benefit cancer
45	TEM8	Binding to PKM2	Stimulation of angiogenesis by binding of Tumor M2-PK released from tumors	Dual roles of PKM2 in cancer metabolism
46	TEPP-46	Binding to PKM2	TEPP-46 and FBP, the allosteric activators that induce PKM2 tetramerization	Posttranslational modifications of pyruvate kinase M2: tweaks that benefit
47	TRIM35	MiR-4417 targets TRIM35 and regulates PKM2 Y105 phosphorylation	Promote proliferation and suppress apoptosis, PKM2 Y105 phosphorylation to promote HCC growth	MiR-4417 targets tripartite Motif-containing 35 (TRIM35) and regulates pyruvate kinase muscle 2 (PKM2) phosphorylation to promote proliferation and suppress apoptosis in hepatocellular carcinoma cells

**Table 3:** Post-translational modification of PKM2 protein and its related biological effects

PTM (post-translational modification) effects of PKM2	
Effects on modified protein	Triggered by
Enzymatic activity, induced	K311-sc
Enzymatic activity, inhibited	Y105-p, Y148-p
Enzymatic activity, regulation	K186-Ub, K206-Ub
Intracellular localization	S37-p, S202-p, T405-gl, S406-gl
Molecular association, regulation	S37-p, Y105-p, S202-p, T405-gl, S406-gl
Phosphorylation	S37-p, T405-gl, S406-gl
Protein conformation	K311-sc, T405-gl, S406-gl
Protein stabilization	T328-p, T454-p
Nuclear localization, regulation	K305-Ac, K433-Ac
Effects on biological processes	Triggered by
Apoptosis, inhibited	T328-p
Apoptosis, induced	C358-Ox, P403-OH, P408-OH
Carcinogenesis, induced	S37-p, T454-p
Cell growth, induced	S37-p, Y105-p, Y148-p, T328-p, T405-gl, S406-gl, T454-p
Transcription, induced	S37-p, T328-p
Transcription, inhibited	S37-p
Post-translational modification	Specific Site
Phosphorylation	S37, Y105, Y148, S202, T454
Acetylation	K305, K433
Hydroxylation and oxidation	P403, P408 and K358
Ubiquitination and sumoylation	K186, K206
Glycosylation	T405, S406
Methylation	R445, R447, R455

## Interaction of PKM2 with other proteins

### Phosphorylation

1. The phosphorylated PKM2 can protect mitochondrial from oxidative stress. At the beginning of the period, ROS can oxidize the Cys358 site of PKM2 when tumor cells are exposed to oxidative stress damage. PKM2 acts as a phosphorylase to phosphorylate its substrate [130]. PKM2 can phosphorylate Bcl2 threonine (Thr69), which prevents the binding of Cul3-based E3 ligase to Bcl2 and subsequent inhibits degradation of Bcl2. Then heat shock protein 90 (HSP90) acts as a bridge, stabilizes and transferres the PKM2-BCL2 complex to Mitochondrial outer membrane, which regulates the ratio of Bax/Bcl2. Liang, Cao et al. believe that mitochondrial function still exists in the background of Warburg effect, and the maintenance of mitochondrial function in this case depends on the binding of PKM2 and Bcl2 on the outer membrane of mitochondria, inhibiting the release of ROS and oxidative stress-induced apoptosis [131].
2. The phosphorylated PKM2 can increase its nuclear localization and promote cell proliferation [55].
  1. PKM2 phosphorylates PAK2 directly on Ser20, Ser141 (phosphorylated but weakly acting) and Ser192/197. PKM2-mediated phosphorylation of Ser192/197 is essential for maintaining PAK2 activity in PDAC cells, promotes HSP90 association with PKM2-PAK2 complex, which prevents the degradation of ubiquitin and protease of PAK2, and ultimately mediates tumor cell invasion, metastasis and cell proliferation [132].
  2. After binding to SAICAR, PKM2 phosphorylates the Thr202, Tyr204 and ERK2 Thr202 of ERK1, which in turn phosphorylates the Ser37 of PKM2. This cascaded phosphorylation pattern can form an activation loop and activate the ERK/MAPK signal pathway [55].
  3. PKM2 phosphorylates tyrosine at position Tyr333 on β-catenin, and phosphorylation of this site is necessary for the binding of β-catenin to the PKM2 Tyr site, the binding of PKM2-β-catenin (Tyr333p) can promote the proliferation and development of tumor cells [124].
  4. PKM2 can phosphorylate Thr11 of histone H3, which regulates the transcription of MYC and CCND1, and promotes the G1-S phase transition of the cell proliferation cycle, as well as Y705 phosphorylating stat3. Phosphorylation of STAT3 can promote the production of CCND1, which can enhance the activity of mitogen activated protein kinase (MAPK) pathway [124].
  5. PKM2 can phosphorylate Tyr118 and phosphorylate Bub3 Tyr27 sites of myosin light chain 2 (MLC2). Phosphorylation of MLC2 can also enhance the activity of mitogen activated protein kinase (MAPK) pathway, and phosphorylated Bub3 can enhance the activity of EGFR pathway [124].
  6. PKM2 can promote the serine at position 202/203 of protein kinase B substrate l (AKT1S1) binding to 14-3-3 to activate the mechanistic target of rapamycin complex l (mTORC l) signal pathway, which will promote tumor growth [127].
  7. In the nucleus of bone marrow cells of leukemia patients, In the nucleus of bone marrow cells of leukemia patients, the researchers found that PKM2 can phosphorylate and activate certain transcription factors, thereby inducing fusion of BCR-ABL genes. In the cytoplasm of these cells, it is characterized by the dissociation of PKM2 tetramer, the formation of dimers, and finally promote the accelerated formation of leukemia in patients [133].
  8. A mode of action similar to the last article 7 still exists in ETV6–NTRK3. PKM2 can phosphorylate and activate certain transcription factors, thereby inducing fusion of Est variant 6 and neurotrophic tyrosine kinase receptor. The result may lead to malignant transformation of some neurogenic tumors. PKM2 in the cytoplasmic dimer form promotes the formation of the Warburg effect [134].
  9. P53 and PKM2 in the nucleus of the cells can phosphorylate each other to form a cascade loop. Although the location of the PKM2 phosphorylated P53 modification site has not been specifically demonstrated, P53 has been shown to phosphorylate the Ser37 site of PKM2. When the tumor cells are under stress, this pattern is activated to protect against external stress in the form of EMT [135].
3. PKM2 not only can act as a phosphorylase to phosphorylate the substrate, but also can be phosphorylated by other phosphorylases at specific sites. The phosphorylation of its tyrosine residue Tyr105 has been found in a variety of solid tumors in humans. For example,
  1. The tyrosine residues Tyr 83, Tyr105, Tyr148, Tyr175, Tyr370, Tyr390 of PKM2 can be directly phosphorylated by fibroblast growth factor receptor 1 (FGFR1), and the binding of phosphorylated PKM2 to FBP inhibits the presence of the tetrameric form of PKM2, results in reducing its PK activity [136].
  2. It has been reported that tripartite motif containing 35 (TRIM35) directly binds to PKM2 to inhibit PKM2 Tyr105 phosphorylation, thereby increasing enzyme activity [137].
  3. ERK1/2 also has phosphorylase activity, which specifically phosphorylates the Ser37 site of PKM2 without phosphorylating PKM1, which provides a binding motif for PKM2 that interacts with peptidyl-proline isomerases, and binding to the transcript of the mitotic gene A1 (PIN1) mediates the entry of PKM2 into the nucleus [138]. When PKM2 enters the nucleus, it can also bind to Oct-4, and combine with many of the above mechanisms to affect the spindle, affecting the cell cycle by affecting cell division [139, 140].
  4. Glycogen synthase kinase 3β (GSK-3β) could phosphorylate Thr-328 of PKM2, and phosphorylation of the PKM2 Thr-328 site is essential for maintaining PKM2 stability and regulating biological functions of glycolysis, mitochondrial respiration, proliferation and apoptosis. HSP90 also has a function to stabilize the PKM2–GSK3β complex in the HSP family [141].
  5. In the cytoplasm of tumor cells, proviral insertion in murine 1ymphomas 2 (PIM2) can also phosphorylate the Thr454 site of PKM2, to mediates PKM2-dependent anaerobic glycolysis, and maintains mitochondrial function in tumor cells [142].
  6. Jun N-terminal kinase 1 (JNK1) phosphorylates the PKM2 threonine residue Thr365 to inhibit PKM2 enzyme activity [143].
  7. PKM2 can be phosphorylated by Fms-related tyrosine kinase 3 (FLT3), Janus kinase 2 (JAK2), protein kinase C (PKC), and the modified PKM2 will exist as a dimer. Promote anaerobic glycolysis and cell proliferation of tumor cells [46, 133].
  8. In addition to phosphorylation of these known sites, there are also some protein molecules with phosphorylation, but the site is not yet clear. Such as A-RAF, after phosphorylation of PKM2, which could regulate the composition ratio between PKM2 dimer and tetramer, and then regulate the glucose metabolism of tumor cells [53]. PKM2 binds to CD44 to inhibit phosphorylation of serine at a certain position in PKM2, inhibit promoting aerobic glycolysis and promoting glucose metabolism to biosynthesis [125].

In addition to phosphorylation, proteins such as protein tyrosine phosphatase 1B (PTP1B) inhibit the phosphorylation of PKM2, and PTP1B inhibits the biological activity of PKM2 by phosphorylating the Tyr105 and Tyr148 site of PKM2 [71].

### Acetylation

Acetylation is another pre-translational modification of PKM2 that reduces the enzymatic activity of PKM2, increases glycolysis intermediates, and supports biosynthesis. One of the important features of the tumor microenvironment is hypoxia. Hypoxia inducible factor-1α (HIF-1α) and hypoxia inducible factor-1β (HIF-1β) are under hypoxic conditions, and the expression will be increased when tumor and normal cells is under hypoxic stress [144]. The same hypoxia transcription factor CBP/P300 can be used as an effector molecule downstream of HIF-1α and HIF-1β, so that it can play an important biological role in the nucleus and cytoplasm. P300 can be used as an acetyltransferase to catalyze exon 10 of PKM2 [145]. PKM2 is acetylated by P300 in Lys433, and the PKM2 Lys433 is acetylated to regulate cell proliferation and transform the biological behavior of PKM2 from cytoplasmic metabolic kinase to nuclear protein kinase activity [146]. Parkin can ubiquitinate the Lys186/206 site of PKM2, and then regulate the ratio of tetramers and dimers of PKM2. In addition, a complex can be formed between the PKM2/P300/PHD3/HIF-1α/HIF-1β in the nucleus, and finally regulate the tumor fine glucose metabolism, O_2_ consumption and CO_2_ production [147]. Acetylation is also present in heat shock cognate protein70 (HSC70) and P300/CBP-associated factor (PCAF), which acetylates the Lys305 site of PKM2. By inhibiting the biological effects of PKM2 by acetylation of the PKM2 Lys305 site, this kind of acetylation behavior mediates the binding of PKM2 to HSC70 and attenuates the degradation of PKM2 via the lysosomal pathway [148]. In addition to HSP70, there is similar acetylation, but the site is unknown. After acetylation of PKM2, HSP70 can acetylate PKM2, which mediates intracellular localization of lysosomes, such maintaining intracellular homeostasis [142]. Table 3 summarizes the various post-translational modification sites mentioned above and the biological effects they exert.

### Other interprotein interactions

The activity of PKM2 is also regulated by many proteins in contact with it, and the binding sites and mechanisms are of action vary. These special regulatory sites are also summarized in Tables 2, 3 and Fig. 6.

(1) Mucin-1 (MUC-1), death-associated protein binding to PKM2 can increase enzyme activity and promote glycolysis; hybrid double yeast technology studies show that PIAS3 (protein inhibitor of activated STATA3) regulates enzyme activity through binding of carboxy terminus to PKM2 to promote glycolysis [149]. (2) O-GlcNAcylation can block the Thr405 and Ser406 sites of PKM2, and the stability of PKM2 in tetrameric form is reduced to enhance the aerobic glycolysis, namely Warburg Effect [150]. (3) The binding of histone demethylation enzyme Jumonji-C (JmjC) domain-containing protein 5 (JMJD5) [151], l-cysteine [152] and HSP40 [153] to PKM2 can reduce the enzyme activity. (4) 5-Amino-4-succinic acid carboxamide imidazole ribonucleotides activate PKM2 by direct contact allosteric regulation. (5) Parkin promotes the ubiquitination of Lys186 and Lys206 sites in PKM2, which inhibits the biological activity of PKM2 and regulates glucose metabolism by promoting ubiquitination of Lys186 and Lys206 sites of PKM2 [154]. (6) PKM2 can bind to SAICAR, and mutation of PKM2 Gly415 site will make PKM2 unable to bind to SAICAR [55]. Prolyl hydroxylase 3 (PHD3) can hydroxylate the Pro403 and Pro408 sites of PKM2 [155]. While PKM2 with the help of methylglyoxal (MG) can glycosylate the Arg399 site of PKM2, the result of which can change [156]. (7) Similarly, in the nucleus PKM2 exon 10 can be methylated by co-activator associated arginine methyltransferase 1 (CARM1), but the specific location is unknown. After being methylated by CARM1, PKM2 can promote aerobic glycolysis and malignant transformation of tumors [157].
Because PKM2 can enter the nucleus to act as a transcriptional regulator, the intermodulation between proteins is also present in the nucleus, and NAD-dependent deacetylase sirtuin6 (SIRT 6) can deacetylate the 433th lysine of PKM2 in the nucleus [158]. It inhibits PKM2 transfer out of the nucleus and inhibits tumor growth. Proline hydroxylation of PKM2 at 403 and 408 by the PHD3 enzyme favors the interaction of PKM2 with the HIF-1α transcription complex, which results in recruitment of P300-acetyltransferase to facilitate the transactivation of HIF target genes.
Although this mode of regulation of intermolecular modification is more common in cells, for PKM2 proteins with three-dimensional structure, the mode of regulation involved is not limited to the modes we mentioned above. For example, for certain protein molecules, it does not have protein kinase activity, but it can bind to a specific domain on PKM2, thereby regulating the biological activity of PKM2 [159]. For example: (1) probable E3 ubiquitin-protein ligase (HERC-1) can bind to the amino acid sequence of paragraphs 406 to 531 of PKM2. HERC-1 can ubiquitinate PKM2 to induce MET processes in tumor cells [160]. (2) SUMO E3-ligase (PIAS-3) can bind to the amino acid sequence of paragraphs 1 to 348 of PKM2. The sumoylation of PKM2 can promote its nuclear localization [161]. (3) Oct-4 can bind to the amino acid sequence of paragraphs 307 to 531 of PKM2. It can work with PKM2 to regulate gene transcription in cells, thereby regulating cell cycle and cell division [161]. (4) Opa can bind to the amino acid sequence of paragraphs 367 to 531 of PKM2. The interaction between OPA and PKM2 can promote the MET process of the free tumor cells that have undergone MET conversion, and then adhere to the metastatic site to invade [162]. (5) This interaction also occurred between HIF-1α/β, HCV, HPV, PANK-4, PML, SOCS-3, TEM-8, etc. protein and PKM2. In addition to promoting the formation of PKM2 dimer, its role is to promote the nuclear localization of PKM2. This in turn induces transcription and translation of downstream genes of PKM2 and exacerbates the malignancy of tumor cells [46, 163–165].
Co-activator-associated arginine methyltransferase 1 (CARM1) also known as PRMT4 methylates specifically the dimeric form of PKM2 at Arg445, Arg447, Arg455 residues in the C domain which is PKM2 exon 10 located. TEPP-46 and FBP, the allosteric activators that induce PKM2 tetramerization limits PKM2 methylation. Importantly, PKM2 activity remains unaltered by methylation; however, methylated PKM2 reprograms the metabolic phenotype toward aerobic glycolysis from oxidative phosphorylation to support tumor cell proliferation, migration, and metastasis [161].

PKM2 can not only exert the activity of PK enzyme in the form of tetramer, but also can enter the nucleus as a transcription factor to mediate the transcription of other genes when convert to dimer, can also regulate each other in the cytoplasm and other proteins. It has an impact on many different biological effects.

## The application of PKM2 in Clinical

Although in this paper we have already talked about the biological effects of PKM2’s many non-canonical PK enzyme activities, PKM2 is ubiquitously expressed in embryogenesis, tissue regeneration and cancer under normal conditions, However, we are most aware of its classical pyruvate kinase activity, and pyruvate kinase activity plays a crucial role in actively proliferating cells [166]. After analyzing the RNASeq dataset for PKM2 in certain tissues, some researchers found that PKM2 is also expressed in some differentiated tissues and non-proliferating cells. PKM2 plays an important role in maintaining the metabolic process of cancer cells. When PKM1 replace PKM2 expressing in tumor cells, the cancer cells will change from mitochondrial function-locked glucose aerobic glycolysis to glucose aerobic oxidation and mitochondrial respiration, and the tumor’s ability of proliferation, metastasis, invasion ability and EMT progression are significantly decreased [167, 168]. There have been many studies pointing out that PKM2 can be highly expressed in lung cancer, liver cancer, glioma, kidney cancer, etc., and there are close relationships with TMN staging, clinical stage, prognosis of patients and malignant degree of tumor, whether distant invasion and metastasis occur, etc. The more popular tumor starvation therapy that has been studied is also targeted at key enzymes of glucose metabolism including PKM2, and it is expected to effectively treat tumors [169].

The pyruvate kinase activity of PKM2 is well known to the public, however, non-canonical PK enzymatic activity of PKM2 plays a more potent role in tumors. In particular, after exon 10 replacing exon 9, it gives PKM2 a more abundant biological function, and migrate the PKM2, a protein that should be localized in the cytoplasm into the nucleus to activate transcription and translation of certain genes. At the same time, PKM2 can also act as a phosphorylase to promote the phosphorylation of a variety of substrates, while also being phosphorylated and acetylated to bind to certain proteins, thereby exerting unexpected functions. The herbal extract, Shikonin, may inhibit PKM2 activity by binding to PKM2 [170]. Recently have discovered PKM2 inhibitor-Compound 3K, researchers have found that injecting this inhibitor into the tail vein of mice after implantation of tumors can inhibit the growth of tumors implanted in mice, causing cell death to exert anticancer activity [171].

**Fig. 7:**
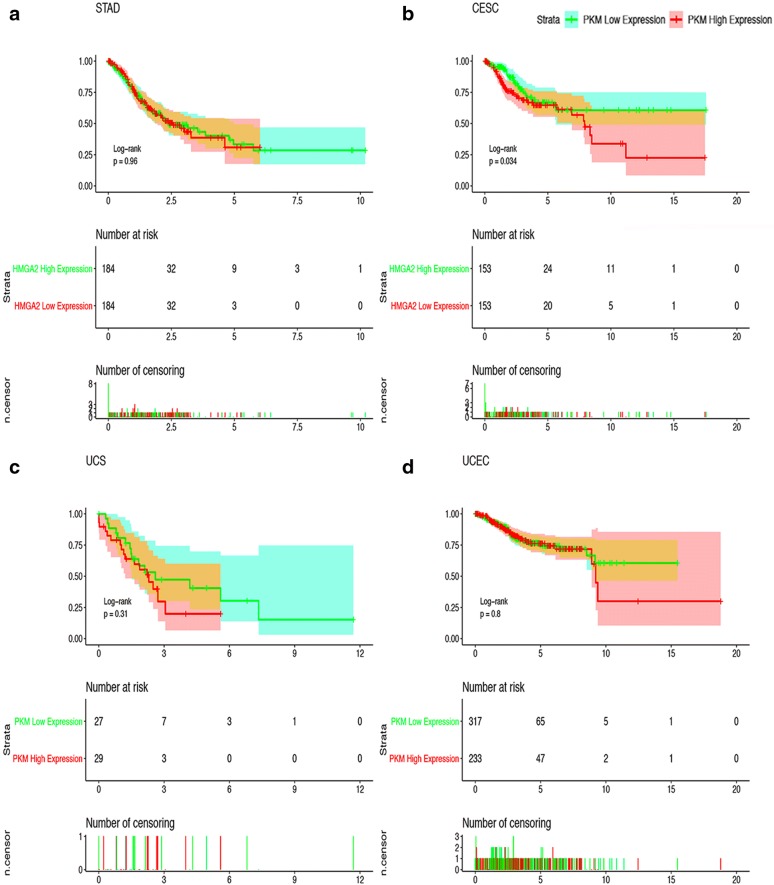
Kaplan–Meier Curves for Survival of Four Most Relevant Cancers. Kaplan–Meier curves for survival of four most relevant cancers according to PKM2 special transcript (NP-002645.3 refer to Fig. 5a and Table 1) expression in cancer tissues. Patients were divided into high and low PKM2 special transcript expression groups using the median value of PKM2 special transcript expression as the cutpoint. Survival analysis and subgroup analysis were performed based on Kaplan–Meier curves. One thing should be pointed out is that in some clinical studies, the researchers found that the effect of contrast PKM2 protein expression was significantly better than the copy number of the comparative mRNA. But considering that there is currently no database on protein expression, we only counted the data in TGCA

Kaplan–Meier curves for survival of four most relevant cancers according to PKM2 special transcript (NP-002645.3 refer to Fig. 5a and Table 1) expression in cancer tissues. Patients were divided into high and low PKM2 special transcript expression groups using the median value of PKM2 special transcript expression as the cutpoint. Survival analysis and subgroup analysis were performed based on Kaplan–Meier curves. One thing should be pointed out is that in some clinical studies, the researchers found that the effect of contrast PKM2 protein expression was significantly better than the copy number of the comparative mRNA. But considering that there is currently no database on protein expression, we only counted the data in TGCA (Fig. 7).

## Outlook

PKM2 not only plays a role of rate-limiting enzyme in glucose metabolism, but also regulates tumor cell energy metabolism reprogramming (EMR) and mitochondrial function Adaptive Mitochondrial Reprogramming (AMR) together with other key enzymes of glucose metabolism. Both the Warburg effect proposed by Warburg and the “post-Warburg effect” proposed by follow-up scholars all predict the important role of PKM2 in tumor development, invasion and metastasis, and diagnosis, treatment and prognosis of tumor patients [172, 173]. I believe that with the deepening of the research on PKM2, the research on its unique biological effects in the cytoplasm and nucleus will prompt researchers including myself to deeply analyze the energy metabolism reprogramming and mitochondrial function Adaptive Mitochondrial Reprogramming of experimental tumor cells. And provide a broader road treatment for tumors.

## Additional files

**Additional file 1: Table S1.** PKM2 related GO terms and KEGG pathways in cancer tissue. GO terms and KEGG pathways with P-values < 0.05 were considered statistically significant. GO: Gene Ontology. KEGG: Kyoto Encyclopedia of Genes and Genomes.

**Additional file 2: Fig. S1.** The co-expressed genes predicted by MEM. Genes that are co-expressed with PKM2 were subsequently identified through MEM. Including 100 datasets including 1694 samples were used to analysis the genes co-expressed with PKM2 in MEM (https://biit.cs.ut.ee/mem/). In Fig.S1, we list 30 genes most closely related to PKM2. The significantly GO terms and KEGG pathways were identified by KOBAS and DAVID which will be listed in Figs. S2 and S3. MEM: Multi Experiment Matrix. DAVID: Database for Annotation, Visualization and Integrated Discovery. KOBAS: KO-Based Annotation System.

**Additional file 3: Fig. S2.** The significantly GO terms identified by DAVID. Three GO terms [biological process (BP), cellular component (CC) and molecular function (MF)] were utilized to identify the enrichment of target genes by DAVID (http://david.abcc.ncifcrf.gov/). The enrichment map of annotation analysis was drawn using Cytoscape (version 3.3.1) (http://www.cytoscape.org/cy3.html). GO: Gene Ontology. DAVID: Database for Annotation, Visualization and Integrated Discovery.

**Additional file 4: Fig. S3.** The significantly KEGG pathways identified by KOBAS. Using the 30 genes predicted by MEM, 34 different KEGG pathways can be enriched by KOBAS (http://kobas.cbi.pku.edu.cn/). These signaling pathways can be roughly divided into six broad categories. The results were generated using the visualization tool in R (version 3.5.3). KOBAS: KO-Based Annotation System. KEGG: Kyoto Encyclopedia of Genes and Genomes. MEM: Multi Experiment Matrix.

**Additional file 5: Fig. S4.** The functional protein association network enriched by STRING. PKM2 and its related proteins can not only constitute a large regulatory network to affect the energy metabolism of tumor cells, but also can form many small regulatory networks to affect different biological activities. The data used was derived from the 100 genes predicted by the MEM database by STRING (https://string-db.org/), which are highly correlated with PKM2.

## Abbreviation

α-KG: alpha-ketoglutarate
AA: amino acid residues
Ac-CoA: acetyl coenzyme A
ADP: adenosine diphosphate
AKT1S1: protein kinase B substrate l
Ala: alanine
AMR: adaptive mitochondrial reprogramming
A-Raf: a family of three serine/threonine-specific protein kinases related to retroviral oncogenes
Arg: arginine
ATP: adenosine triphosphate
β-cat: β-catenin
BCL2: B-cell lymphoma-2
Bub: budding uninhibited by benzimidazoles
CARM1: co-activator associated arginine methyltransferase 1
CBP: CREB-binding protein
CCND1: cyclin D
EGF: epidermal growth factor
EGFR: epidermal growth factor receptor
EMT: epithelial–mesenchymal transition
EMR: energy metabolism reprogramming
ERKl/2: extracellular signal-regulated kinase 1/2
Etv6–NTRK3: Est variant 6 and neurotrophic tyrosine kinase receptor
FADH2: flavine adenine dinucleotide
FAO: fatty acid oxidation
F6P: fructose 6-phosphate
FBP: fructose 1,6-bisphosphate
FLT3: Fms-related tyrosine kinase 3
FGFR1: fibroblast growth factor receptor type 1
Glu: glutamic
Gly: glycine
GSK-3β: glycogen synthase kinase 3β
G6P: glucose 6-phosphate
HCV: Hepatitis C virus
HERC-1: E3 ubiquitin-protein ligase
HIF-1α: hypoxia inducible factor-1α
HIF-1β: hypoxia inducible factor-1β
HK: hexokinase
HPV: human papillomavirus
HSC70: heat shock cognate protein70
hnRNPA1: heterogeneous nuclear ribonucleoprotein A1
hnRNPA2: heterogeneous nuclear ribonucleoprotein A2
HSP: heat shock protein
IDH2: isocitrate dehydrogenase 2
Iso: isoleucine
JAK2: janus kinase 2
JNK: c-Jun N-terminal kinases
JmjC: Jumonji-C
JMJD5: Jumonji-C (JmjC) domain-containing protein 5
LDH: lactate dehydrogenase
Leu: leucine
Lys: lysine
MAPK: mitogen activated protein kinase
MAPKK5/MEK5: mitogen activated protein kinase kinase 5
MG: methylglyoxal
MLC2: myosin light chain 2
MiR/miRNA: microRNA
MUC-1: Mucin-1
mTORC l: mammalian rapamycin target protein sensitive complex 1
NADH: nicotinamide adenine dinucleotide
NADPH: triphosphopyridine nucleotide
NEK2: never in mitosis (NIMA)-related kinase 2
NLS: nuclear localization sequence
Oct 4: octamer-binding transcription factor 4
O-Glc: O-GlcNAcylation/acetylglucosamine
Opa: outer membrane proteins involved in gonococcal
OXPHOS: oxidative phosphorylation
PAK2: serine/threonine-protein kinase 2
PANK4: pantothenate kinase 4
PCAF: P300/CBP-associated factor
PKC: protein kinase C
PEP: phosphoenol pyruvate
PFK: phosphofructokinase
PGM: phosphoglycerate mutase
PEP: phosphoenolpyruvate
pH: Hydrogen Ion Concentration
PHD3: prolyl hydroxylase 3
Phe: phenylalanine
PIAS3: protein inhibitor of activated STATA3
PIM2: proviral insertion in murine 1ymphomas 2
PIN1: mitotic gene A1
PHGDH: phosphoglycerate dehydrogenase
PKB: protein kinase B/2
PKL: liver isoform of pyruvate kinase
PKLR: liver and red blood cell pyruvate kinase
PKM1: M1 isoform of pyruvate kinase
PKM2: M2 isoform of pyruvate kinase
PK: pyruvate kinase
PKR: red blood cell isoform of pyruvate kinase
PML: promyelocytic leukemia
PPP: pentose phosphate pathway
Pro: proline
PTP1B: protein tyrosine phosphatase 1B
PTB: polypyrimidine tract binding protein
PTBP1: polypyrimidine bundle binding protein 1
PTM: post-translational modification
PYP: polyol pathway
REDOX: oxidation–reduction
ROS: reactive oxygen species
SAICAR: succinylaminoimidazolecarboxamide ribose-50 phosphate SDH succinate dehydrogenase
SDH: succinate dehydrogenase
Ser: serine
SRSF-3: serine/arginine-rich protein-specific kinase
SIRT 6: NAD-dependent deacetylase sirtuin6
SOCS 3: suppressor of cytokine signaling 3
STAT3: signal transducers and activators of transcription 3
T3: thyroid hormone T3
TCA: tricarboxylic acid cycle
TEM8: human tumor endothelial factor 8
T3: thyroid hormone 3,3,5-triiodo-L-thyronine
Thr: threonine
TRIM35: tripartite motif containing 35
Tyr: tyrosine
UAP: uronic acid pathway
VEGF: vascular endothelial growth factor
