# Correction to: PKM2, function and expression and regulation

**DOI:** 10.1186/s13578-019-0321-z

**Published:** 2019-07-17

**Authors:** Ze Zhang, Xinyue Deng, Yuanda Liu, Yahui Liu, Liankun Sun, Fangfang Chen

**Affiliations:** 1 Department of General Surgery, The First Hospital of Jilin University, Changchun, 130021 China; 2 Department of Pathophysiology, College of Basic Medical Sciences, Jilin University, Changchun, 130021 China; 3 Department of Gastrointestinal Surgery, The Second Hospital of Jilin University, Changchun, 130041 China; 4 Department of Gastrointestinal Colorectal and Anal Surgery, China-Japan Union Hospital of Jilin University, Changchun, 130021 China

## Correction to: Cell Biosci (2019) 9:52 10.1186/s13578-019-0317-8

In the publication of this article [1], there is an error in Fig. 7a and 7b. This has now been included in this correction.

The error in Figure 7a and 7b:

HMGA2 High Expression

HMGA2 Low Expression

Should instead read:

PKM High Expression

PKM Low Expression

The corrected Fig. 7 is given here.

**Fig. 7:**
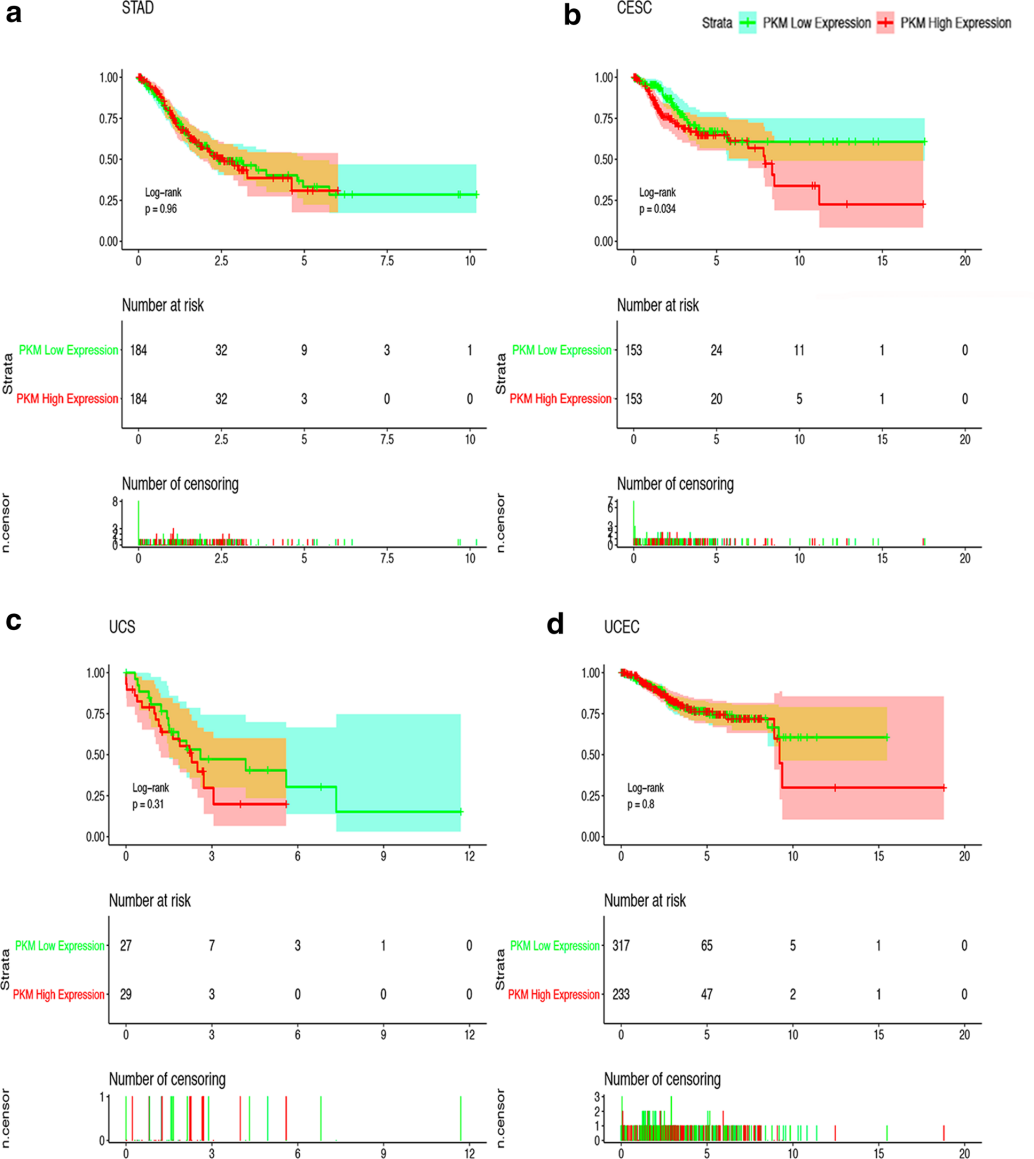
Kaplan–Meier Curves for Survival of Four Most Relevant Cancers. Kaplan–Meier curves for survival of four most relevant cancers according to PKM2 special transcript (NP-002645.3 refer to Fig. 5a and Table 1) expression in cancer tissues. Patients were divided into high and low PKM2 special transcript expression groups using the median value of PKM2 special transcript expression as the cutpoint. Survival analysis and subgroup analysis were performed based on Kaplan–Meier curves. One thing should be pointed out is that in some clinical studies, the researchers found that the effect of contrast PKM2 protein expression was significantly better than the copy number of the comparative mRNA. But considering that there is currently no database on protein expression, we only counted the data in TGCA
