# Supplementary material for: PKM2, function and expression and regulation
Source: Cell Biosci. 2019 Jun 26;9:52. doi: 10.1186/s13578-019-0317-8 (PMC6595688; doi:10.1186/s13578-019-0317-8)
Supplement: Supplementary file 1 — Additional file 1: Table S1. PKM2 related GO terms and KEGG pathways in cancer tissue. GO terms and KEGG pathways with P-values < 0.05 were considered statistically significant. GO: Gene Ontology. KEGG: Kyoto Encyclopedia of Genes and Genomes. [file 13578_2019_317_MOESM1_ESM.docx]

**Table S1. PKM2 related GO terms and KEGG pathways in cancer tissue**

| **GO ID** | **%** | **P-value** | **FDR** | **GO terms** |
| --- | --- | --- | --- | --- |
| GO:0006096  GO:2001241  GO:0050821  GO:0006094  GO:0006487  GO:0070062  GO:0016020  GO:0043209  GO:0005886  GO:0005925  GO:0008250  GO:0070688  GO:0030017 | 13.33333333  4.44444444  6.66666667  4.44444444  4.44444444  42.2222222  22.2222222  11.1111111  22.2222222  11.1111111  4.444444444  4.444444444  4.444444444 | 2.59E-08  0.039965804  0.041809475  0.052939053  0.059361552  5.89E-06  3.08E-04  4.97E-04  0.008707226  0.010473716  0.022649786  0.022649786  0.022649786 | 3.11E-05  38.69285038  40.09039011  47.92498355  52.00690202  0.006367996  0.332607001  0.535357571  9.018702196  10.7556975  21.93313089  21.93313089  21.93313089 | glycolytic process  positive regulation of extrinsic apoptotic signaling pathway in absence of ligand  protein stabilization  gluconeogenesis  protein N-linked glycosylation  extracellular exosome  membrane  myelin sheath  plasma membrane  focal adhesion  oligosaccharyltransferase complex  MLL5-L complex  sarcomere |
|  |  |  |  |  |
| **KEGG ID** | **R-Value** | **P-value** |  | **KEGG pathways** |
| hsa01200  hsa00010  hsa01230  hsa04066  hsa00030  hsa01100  hsa04810  hsa04390  hsa00051  hsa05016  hsa04510  hsa04961  hsa05130  hsa04144  hsa04721  hsa05131  hsa05100  hsa05132  hsa03015  hsa04611  hsa04114  hsa04728  hsa05160  hsa04530  hsa05012  hsa04261  hsa04145  hsa04921  hsa04141  hsa04022  hsa04360  hsa05164 | 3.76E-10  8.77E-10  1.26E-07  2.94E-05  3.35E-05  5.15E-05  0.00028567  0.002497448  0.003057228  0.003823009  0.004016851  0.004501211  0.005617044  0.006291897  0.006291897  0.006291897  0.008410374  0.009585038  0.010338557  0.016338102  0.016338102  0.017326094  0.017326094  0.018065912  0.018065912  0.019042671  0.019771301  0.01977447  0.020508505  0.020508505  0.021243048  0.021243048 | 4.70E-12  2.19E-11  4.72E-09  1.47E-06  2.09E-06  3.86E-06  2.50E-05  0.000249745  0.000343938  0.000477876  0.000552317  0.000675182  0.00091277  0.001118958  0.00118497  0.001258379  0.001787205  0.002156634  0.002455407  0.004222173  0.004288752  0.004768424  0.004981252  0.005419859  0.005645598  0.006188868  0.006672814  0.006921064  0.007603344  0.007690689  0.008497219  0.008497219 |  | Carbon metabolism  Glycolysis / Gluconeogenesis  Biosynthesis of amino acids  HIF-1 signaling pathway  Pentose phosphate pathway  Metabolic pathways  Regulation of actin cytoskeleton  Hippo signaling pathway  Fructose and mannose metabolism  Huntington's disease  Focal adhesion  Endocrine and other factor-regulated calcium reabsorption  Pathogenic Escherichia coli infection  Endocytosis  Synaptic vesicle cycle  Shigellosis  Bacterial invasion of epithelial cells  Salmonella infection  mRNA surveillance pathway  Platelet activation  Oocyte meiosis  Dopaminergic synapse  Hepatitis C  Tight junction  Parkinson's disease  Adrenergic signaling in cardiomyocytes  Phagosome  Oxytocin signaling pathway  Protein processing in endoplasmic reticulum  cGMP-PKG signaling pathway  Axon guidance  Influenza A |

Abbreviation: GO: Gene Ontology. KEGG: Kyoto Encyclopedia of Genes and Genomes.
