# Supplementary figures and images for: PKM2, function and expression and regulation
Source: Cell Biosci. 2019 Jun 26;9:52. doi: 10.1186/s13578-019-0317-8 (PMC6595688; doi:10.1186/s13578-019-0317-8)

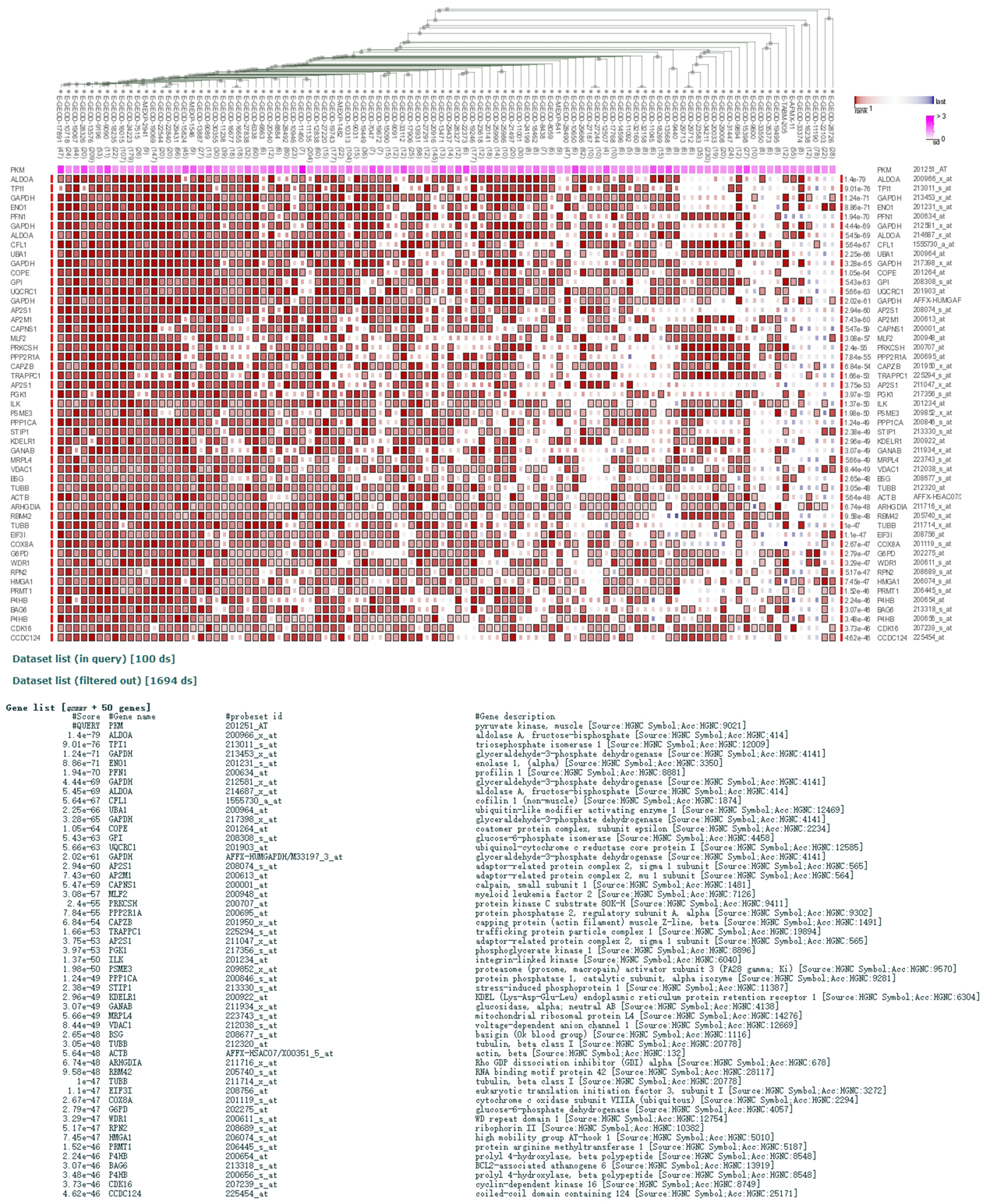

Supplement: Supplementary file 2 — Additional file 2: Fig. S1. The co-expressed genes predicted by MEM. Genes that are co-expressed with PKM2 were subsequently identified through MEM. Including 100 datasets including 1694 samples were used to analysis the genes co-expressed with PKM2 in MEM (https://biit.cs.ut.ee/mem/). In Fig.S1, we list 30 genes most closely related to PKM2. The significantly GO terms and KEGG pathways were identified by KOBAS and DAVID which will be listed in Figs. S2 and S3. MEM: Multi Experiment Matrix. DAVID: Database for Annotation, Visualization and Integrated Discovery. KOBAS: KO-Based Annotation System. [file 13578_2019_317_MOESM2_ESM.tif]

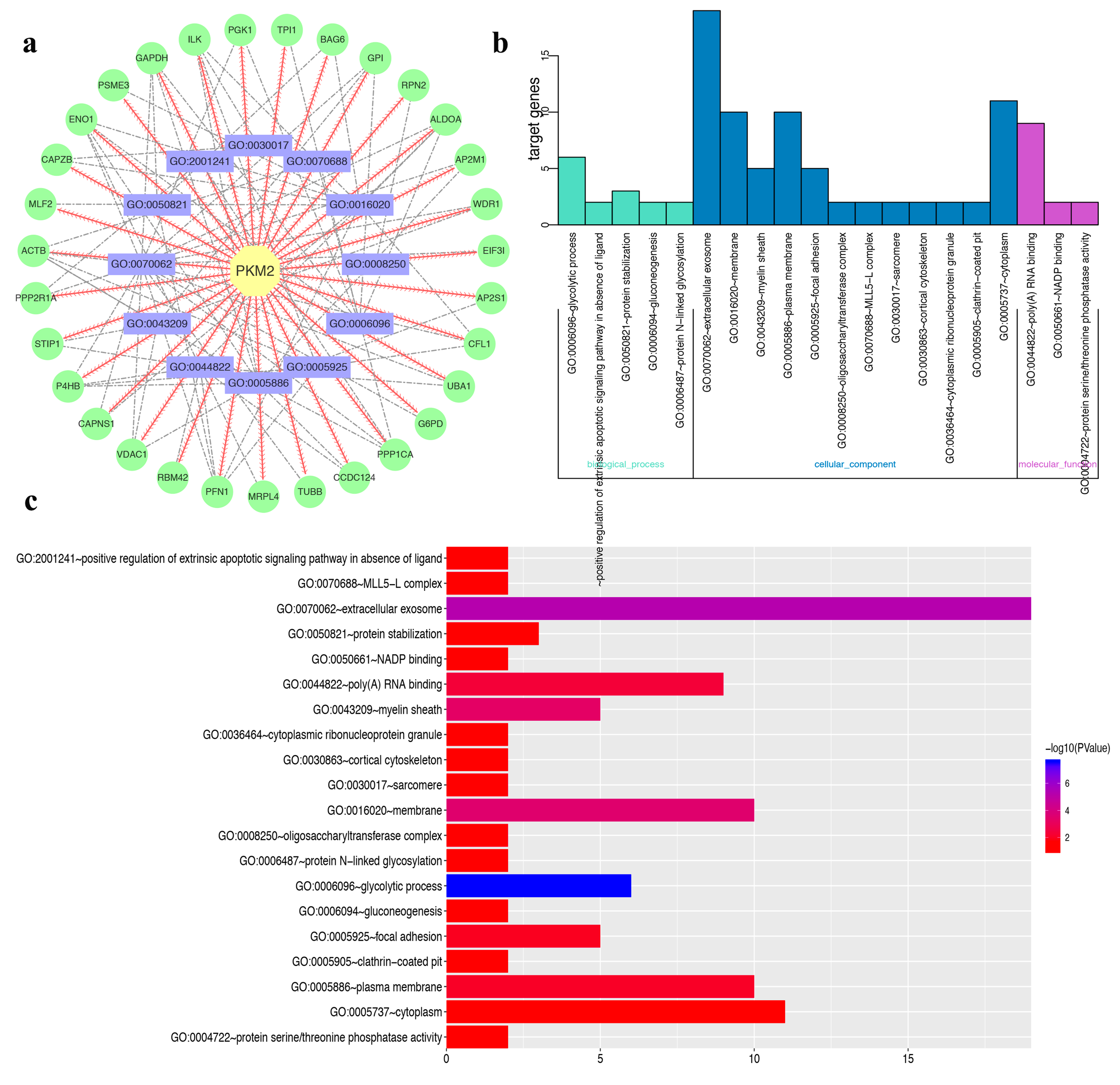

Supplement: Supplementary file 3 — Additional file 3: Fig. S2. The significantly GO terms identified by DAVID. Three GO terms [biological process (BP), cellular component (CC) and molecular function (MF)] were utilized to identify the enrichment of target genes by DAVID (http://david.abcc.ncifcrf.gov/). The enrichment map of annotation analysis was drawn using Cytoscape (version 3.3.1) (http://www.cytoscape.org/cy3.html). GO: Gene Ontology. DAVID: Database for Annotation, Visualization and Integrated Discovery. [file 13578_2019_317_MOESM3_ESM.tif]

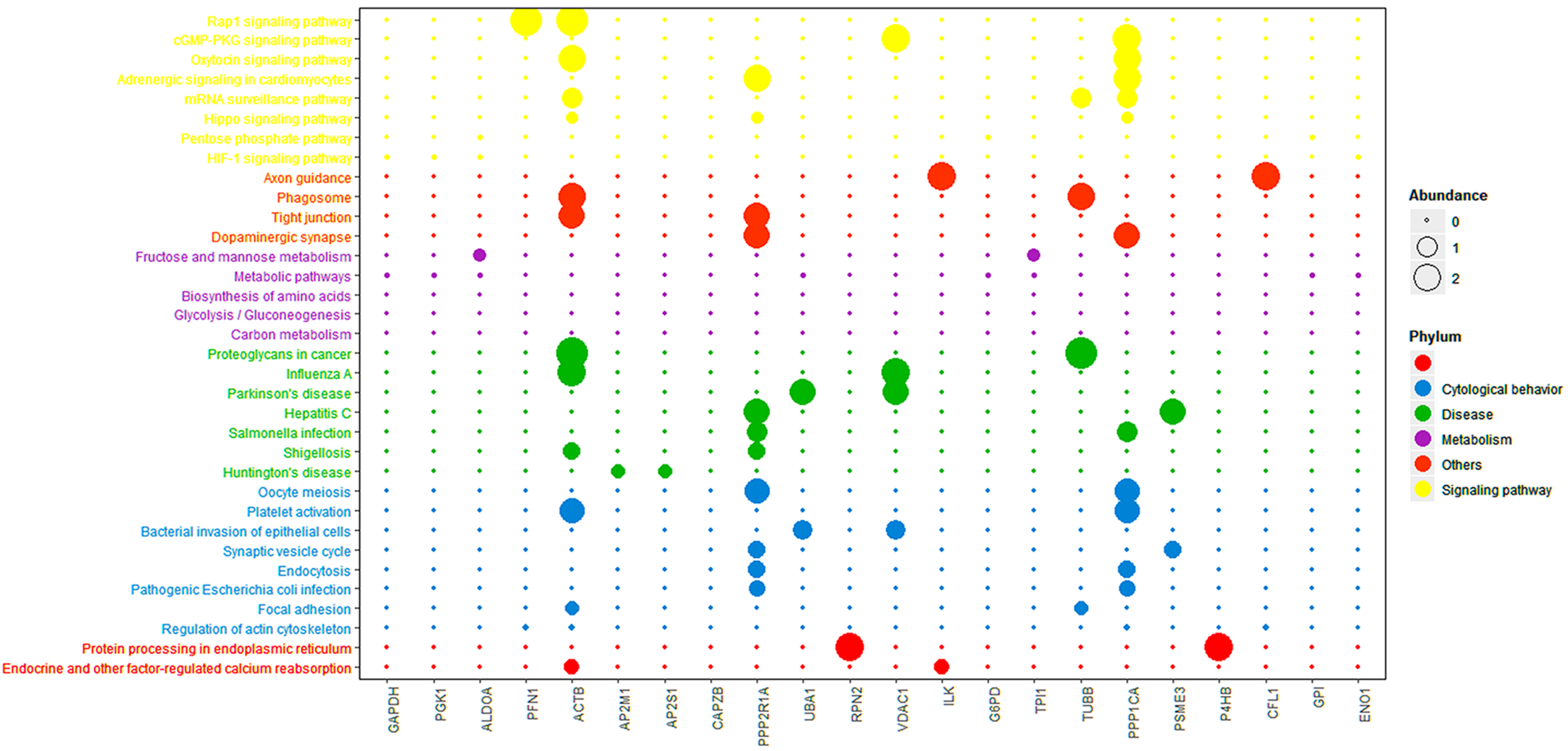

Supplement: Supplementary file 4 — Additional file 4: Fig. S3. The significantly KEGG pathways identified by KOBAS. Using the 30 genes predicted by MEM, 34 different KEGG pathways can be enriched by KOBAS (http://kobas.cbi.pku.edu.cn/). These signaling pathways can be roughly divided into six broad categories. The results were generated using the visualization tool in R (version 3.5.3). KOBAS: KO-Based Annotation System. KEGG: Kyoto Encyclopedia of Genes and Genomes. MEM: Multi Experiment Matrix. [file 13578_2019_317_MOESM4_ESM.tif]

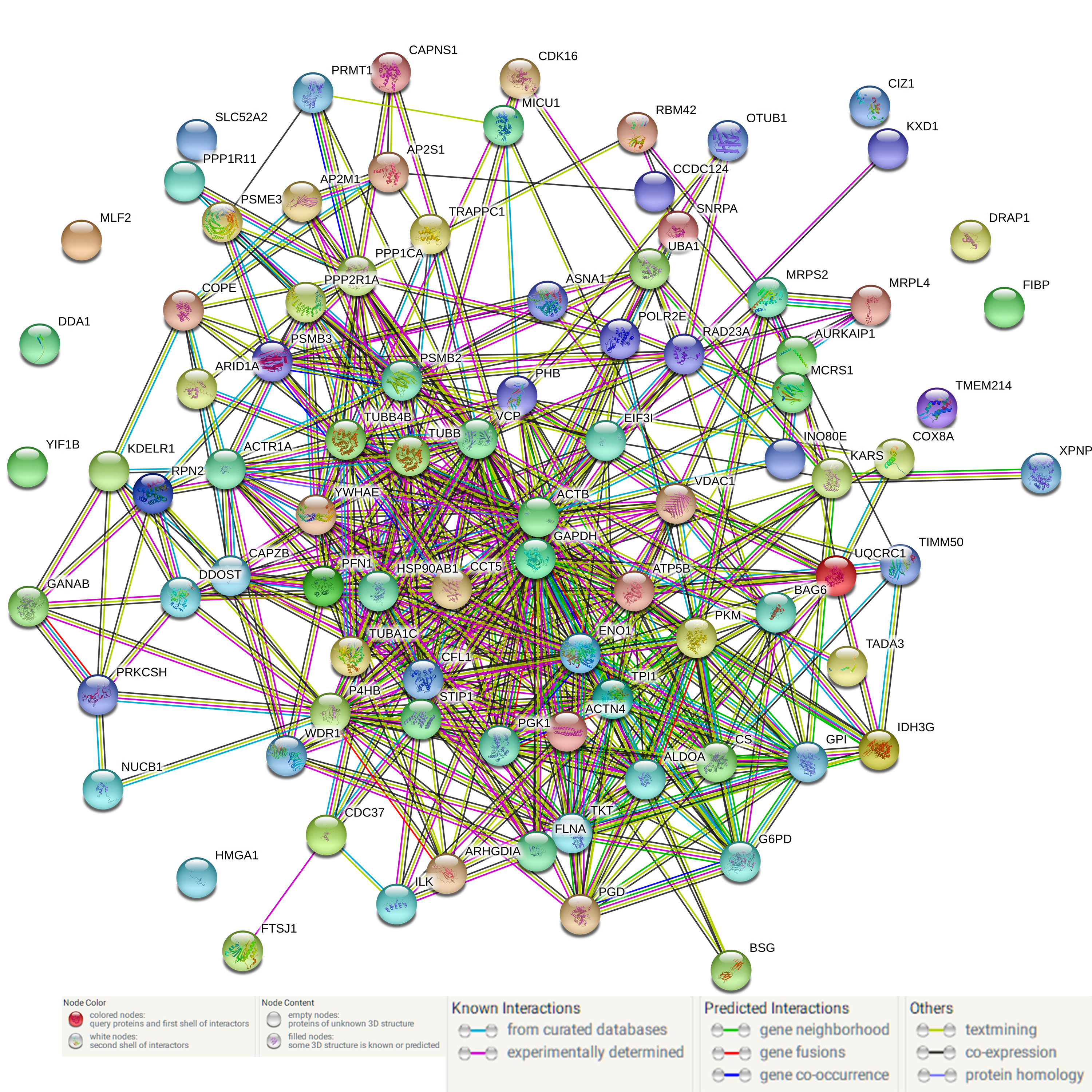

Supplement: Supplementary file 5 — Additional file 5: Fig. S4. The functional protein association network enriched by STRING. PKM2 and its related proteins can not only constitute a large regulatory network to affect the energy metabolism of tumor cells, but also can form many small regulatory networks to affect different biological activities. The data used was derived from the 100 genes predicted by the MEM database by STRING (https://string-db.org/), which are highly correlated with PKM2. [file 13578_2019_317_MOESM5_ESM.tif]
